# Costs-effectiveness and cost components of pharmaceutical and non-pharmaceutical interventions affecting antibiotic resistance outcomes in hospital patients: a systematic literature review

**DOI:** 10.1136/bmjgh-2023-013205

**Published:** 2024-02-29

**Authors:** Kasim Allel, María José Hernández-Leal, Nichola R Naylor, Eduardo A. Undurraga, Gerard Joseph Abou Jaoude, Priyanka Bhandari, Ellen Flanagan, Hassan Haghparast-Bidgoli, Koen B Pouwels, Laith Yakob

**Affiliations:** 1 Disease Control Department, London School of Hygiene & Tropical Medicine, London, UK; 2 Institute for Global Health, University College London, London, UK; 3 Department of Health and Community Sciences, University of Exeter, Exeter, UK; 4 Department of Community, Maternity and Paediatric Nursing, University of Navarra, Pamplona, Spain; 5 Millennium Nucleus on Sociomedicine, Santiago, Chile; 6 Department of Health Services Research and Policy, London School of Hygiene & Tropical Medicine, London, UK; 7 HCAI, Fungal, AMR, AMU & Sepsis Division, UK Health Security Agency, London, UK; 8 Escuela de Gobierno, Pontificia Universidad Catolica de Chile, Santiago, Chile; 9 CIFAR Azrieli Global Scholars program, Canadian Institute for Advanced Research, Toronto, Ontario, Canada; 10 Nuffield Department of Population Health, University of Oxford, Oxford, UK; 11 The National Institute for Health Research Health Protection Research Unit in Healthcare Associated Infections and Antimicrobial Resistance, University of Oxford, Oxford, UK

**Keywords:** Public Health, Control strategies, Prevention strategies, Systematic review, Infections, diseases, disorders, injuries

## Abstract

**Introduction:**

Limited information on costs and the cost-effectiveness of hospital interventions to reduce antibiotic resistance (ABR) hinder efficient resource allocation.

**Methods:**

We conducted a systematic literature review for studies evaluating the costs and cost-effectiveness of pharmaceutical and non-pharmaceutical interventions aimed at reducing, monitoring and controlling ABR in patients. Articles published until 12 December 2023 were explored using EconLit, EMBASE and PubMed. We focused on critical or high-priority bacteria, as defined by the WHO, and intervention costs and incremental cost-effectiveness ratio (ICER). Following Preferred Reporting Items for Systematic review and Meta-Analysis guidelines, we extracted unit costs, ICERs and essential study information including country, intervention, bacteria-drug combination, discount rates, type of model and outcomes. Costs were reported in 2022 US dollars ($), adopting the healthcare system perspective. Country willingness-to-pay (WTP) thresholds from Woods *et al* 2016 guided cost-effectiveness assessments. We assessed the studies reporting checklist using Drummond’s method.

**Results:**

Among 20 958 articles, 59 (32 pharmaceutical and 27 non-pharmaceutical interventions) met the inclusion criteria. Non-pharmaceutical interventions, such as hygiene measures, had unit costs as low as $1 per patient, contrasting with generally higher pharmaceutical intervention costs. Several studies found that linezolid-based treatments for methicillin-resistant *Staphylococcus aureus* were cost-effective compared with vancomycin (ICER up to $21 488 per treatment success, all 16 studies’ ICERs<WTP). Infection control measures such as hand hygiene and gown usage (ICER=$1160/QALY or $4949 per ABR case averted, all ICERs<WTP) and PCR or chromogenic agar screening for ABR detection were highly cost-effective (eg, ICER=$1206 and $1115 per life-year saved in Europe and the USA). Comparisons were hindered by within-study differences.

**Conclusion:**

Robust information on ABR interventions is critical for efficient resource allocation. We highlight cost-effective strategies for mitigating ABR in hospitals, emphasising substantial knowledge gaps, especially in low-income and middle-income countries. Our study serves as a resource for guiding future cost-effectiveness study design and analyses.

**PROSPERO registration number** CRD42020341827 and CRD42022340064

**WHAT IS ALREADY KNOWN ON THIS TOPIC**

- Pharmaceutical and non-pharmaceutical interventions play a crucial role in global antibiotic resistance (ABR) control and prevention.
- There is a paucity of data on the comprehensive health economic costs and outcomes, with most existing literature reviews targeting specific interventions, such as antimicrobial stewardship.

**WHAT THIS STUDY ADDS**

- We synthesised global literature on unit costs and effectiveness of pharmaceutical and non-pharmaceutical interventions among hospitalised patients.
- Despite substantial heterogeneity and some studies lacking fundamental cost and methodological considerations (eg, discounting, risk scenarios and outcomes including hospital stay or mortality), we identified several interventions with robust evidence supporting their benefit, translated into cost or utility-adjusted life years averted.

**HOW THIS STUDY MIGHT AFFECT RESEARCH, PRACTICE OR POLICY**

- Our results aid decision-making by guiding the allocation of scarce resources for combating ABR in hospitals.
- Further investigations, empirical and methodological, are essential to advance the economic evaluation of interventions to progress toward optimising antibiotic usage and reducing ABB rates in hospitals, especially in low-income and middle-income countries.

## Introduction

Antibiotic resistance (ABR) causes an enormous burden on health systems and the global economy.^1–4^ According to a recent study by the Global Burden of Disease, approximately 1.27 million deaths worldwide in 2019 were attributable to ABR if all ABR infections were replaced by drug-susceptible infections.^2^ The World Bank projects an annual global cost of up to $3.4 trillion by 2030 if no action is taken.^5^ The US Centers for Disease Control and Prevention has estimated an annual impact of ABR infections on healthcare and societal costs of approximately $25 billion in the USA.^6^ While these estimates are based on limited data, they underscore the severity of ABR. Setting-specific and population-specific strategies designed to alleviate ABR burden by reducing antibiotic usage and resistance transmission are crucial to reducing loss of life and minimising costs.

Economic evaluations provide critical insights for decision-makers about how to allocate limited healthcare budgets to optimise overall population health. Despite finances underlying healthcare management strategy,^7^ economic evaluations of alternative interventions are surprisingly scarce. Those that are conducted often fail to capture key costs and outcomes required to decide whether to retain the status quo or take up a novel alternative. For example, daptomycin was the first cyclic lipopeptide with demonstrable activity against vancomycin-resistant gram-positive pathogens. It was shown to have equivalent clinical effectiveness in treating complicated skin infections compared with semi-synthetic penicillin while resulting in shorter hospital stays for patients.^8^ Even in this economic evaluation of daptomycin compared with penicillin, however, treatment costs were not explicitly considered, so ambiguity remained over daptomycin’s economic dominance.

Studies synthesising the economic evidence base for alternative ABR-mitigating strategies are equally rare. Previous reviews reporting on economic evaluations of interventions to prevent and control ABR are limited.^9–12^ Naylor *et al* reviewed the cost-effectiveness of antimicrobial stewardship programmes, with estimates ranging from $540 in inpatient net savings to $24 231 for each prevented death.^9^ In a similar review, Huebner *et al* found that targeted control of appropriate antimicrobial agents could save up to $2403 in total antibiotic costs per 100 patient-days.^12^ Niewiadomska *et al* reviewed mathematical modelling studies on the population-level transmission of ABR; however, only 9% of reviewed models included details of cost-effectiveness analyses.^10^ Among these, universal surveillance and decolonisation programmes were cost-saving in patients with methicillin-resistant *Staphylococcus aureus* (MRSA) infections.^12^ Wilton *et al*’s review of studies of the (cost-)effectiveness of interventions for ABR control, including restricting antimicrobials use, prescriber education, use of guidelines for ABR, combination therapies and vaccination,^11^ highlighted the paucity of evidence as a key limitation in delivering definitive and actionable recommendations for ABR control.^11^

Our study aims to systematically synthesise the economic evidence for pharmaceutical and non-pharmaceutical interventions to reduce, monitor and control ABR of critical or high-priority bacteria, as defined by the WHO, including colonisation, infection and antibiotic usage, in hospital settings globally from a health system or payer perspective.^13^ To our knowledge, this is the first review contrasting all available economic and effectiveness components for both intervention types while focusing on key ABR pathogens. By formalising costs and effectiveness for both intervention types in hospital patients, we offer a comprehensive synthesis of ABR interventions conducted within healthcare settings.

## Methods

We conducted a systematic literature review of the costs and cost-effectiveness of pharmaceutical and non-pharmaceutical interventions to reduce, monitor and control ABR levels in hospitalised patients. We followed the Preferred Reporting Items for Systematic Reviews and Meta-Analyses (PRISMA)^14^ and the ISPOR (The Professional Society for Health Economics and Outcomes Research)^15^ guidelines, and our study was prospectively registered with PROSPERO.^14^ The search was conducted on EconLit, EMBASE and PubMed concluding on 12 December 2023.

### Search strategy

We used three key concepts to perform our literature search: (1) ‘Interventions for antibiotic resistance’, (2) ‘Hospital’ and (3) ‘Cost-effectiveness and Economic evaluation’. Economic evaluation filters from InterTASC Information Specialists’ Sub-Group search filters were used to capture the cost-effectiveness aspect of the search. The final literature search strategy and details of studies from the initial screening are presented in online supplemental tables SM1–4.

10.1136/bmjgh-2023-013205.supp1 Supplementary data

### Study selection—inclusion and exclusion criteria

We followed the Patient Population, Intervention, Comparator, Outcome, Setting, Timing (PICOST) framework to present our inclusion and exclusion criteria^16^ (online supplemental tables SM1 and 2). Titles and abstracts of identified articles were screened using Rayyan (https://www.rayyan.ai) by two reviewers for eligibility, and a third reviewer checked them for final inclusion. We contrasted our results with the ‘ASReview’ tool for potential misclassification.^17^ The study population was limited to hospital settings; community settings and acquired infections were excluded. We did not restrict our search by language and years. Studies were included only if the intervention targeted antibiotic-resistant bacterial pathogens listed as critical or high priority by the WHO^18^ (online supplemental table SM3). Bacterial pathogens not on the WHO’s list were excluded. Pharmaceutical interventions were defined as those that directly involved the use of medication, while all other interventions were classified as non-pharmaceutical. Economic evaluations included only complete evaluations (eg, cost-effectiveness, cost-utility, cost-benefit) and were defined as a comparative analysis of the costs and reported the effectiveness of alternative programmes, following Drummond *et al.* 19 Only evaluations using a healthcare or payer perspective were included; very few studies used a societal perspective (n=2). While both perspectives are similar, the healthcare perspective focuses on the costs incurred by providers in delivering medical care and health services to patients and the payer perspective includes the financial aspects of healthcare from the viewpoint of the organisation that funds or reimburses costs to providers. Conference abstracts, editorials and systematic literature reviews were excluded. Papers had to present measures of costs and an incremental cost-effectiveness ratio ‘ICER’ or incremental net monetary and health benefit analyses (ie, a comparison between strategies presenting an ICER).

### Data extraction

We extracted study characteristics and outcomes, including unit costs, effectiveness and cost-effectiveness rates following the Campbell and Cochrane Economic Methods group and a recent protocol for economic appraisal to address ABR which includes specific guidance on reporting health economic data in systematic reviews.^13 20^ For study characteristics, we retrieved the study’s year, author, title, perspective, country, currency, pathogen, intervention, comparator, type of economic evaluation, source of effectiveness data, source of costing and primary outcome. Implementation costs, such as training, were excluded. We also extracted information on the analytical model used, time horizon, discount rate, measure of effectiveness, results of the base-case analysis (eg, ICER) and sensitivity analyses (eg, univariate or multivariate analyses and parameter effects on outcomes). Costs were first converted to US dollars (using currency-specific exchange rates) and inflated to 2022 US dollars based on Gross Domestic Product deflators.^21^ We used the reported costs year, or, if absent, using the publication year instead for exchange rate conversion and subsequent inflation.

### Data synthesis and analysis

We summarise the included data by providing disaggregated unit costs and effectiveness per study and intervention type (pharmaceutical and non-pharmaceutical). Cost-effectiveness estimates were primarily characterised as ICER, including (1) $/(quality-adjusted life-years ‘QALY’ gained), (2) $/(disability-adjusted life-years ‘DALYs’ gained), (3) $/ABR infection averted or (4) $/life-year gained. A dominant strategy refers to a scenario where the incremental cost of the intervention is less than the comparator, and the incremental efficacy is greater than the comparator. Willingness-to-pay (WTP) thresholds per efficiency outcomes were also included, if provided. We identified the gap between individuals’ WTP and the intervention’s real cost-effectiveness to determine the feasibility of the programme in the setting where it was evaluated. Cost-effectiveness thresholds, based on countries' opportunity costs, were employed for strategy comparative purposes and to define resource gaps following Woods *et al.* 22

### Assessment of quality of reporting and risk of bias

We used Drummond *et al*’s checklist for assessing economic evaluations.^23^ The checklist comprises 10 questions for evaluating reporting quality in economic evaluations, assigning a 1 (or 0) to each question if the article included the safeguard (online supplemental table SM5). The aggregate results provided an economic reporting quality appraisal of below average (1–7 points), average (8 points), and above average (9–10 points).

Microsoft Excel was used to create a database of the study characteristics, unit costs and appraisal of studies following the checklist (see https://bit.ly/SR_amrCEingredients).

### Patient and public involvement

The patients and the public were not involved in the design, conduct, or reporting of our research.

## Results

### Study identification and selection

**Figure 1:**
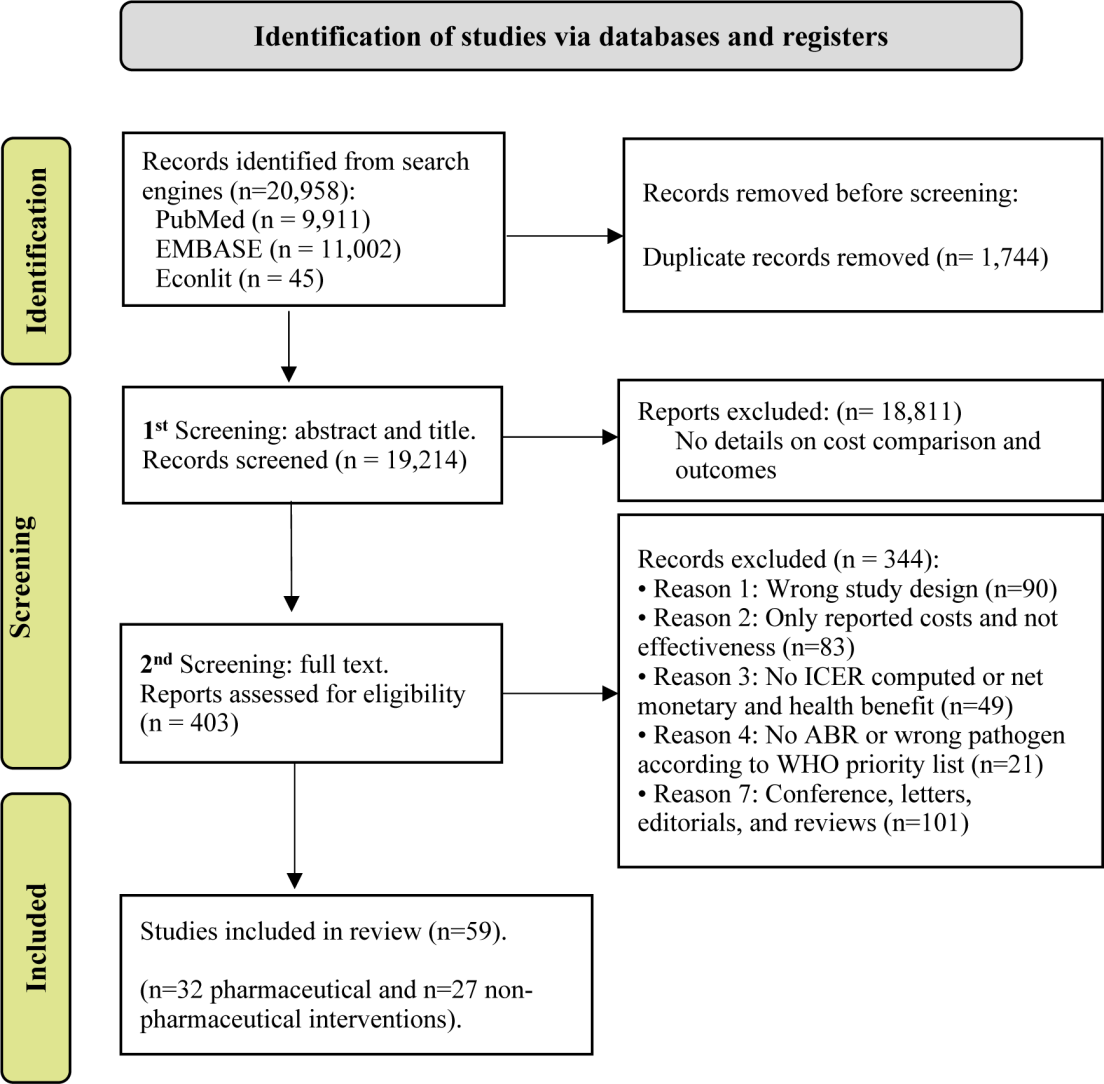
Preferred Reporting Items for Systematic review and Meta-Analysis flowchart for the inclusion and exclusion of relevant studies. ‘n’ stands for the number of articles included/excluded at each stage. ABR, antibiotic resistance; ICER, incremental cost-effectiveness ratio. Source: Moher *et al* 2009.

Figure 1 describes the PRISMA chart for the results of our literature review. We found 20 958 articles in EconLit, EMBASE and PubMed, of which 1744 were duplicated. We excluded 18 811 records due to not fulfilling our inclusion criteria (figure 1). Finally, 403 studies were assessed for full eligibility and 59 (32 on pharmaceutical and 27 on non-pharmaceutical interventions) presented a complete cost-effectiveness analysis and were included in our analytical sample.

### Characterisation of studies included

**Figure 2:**
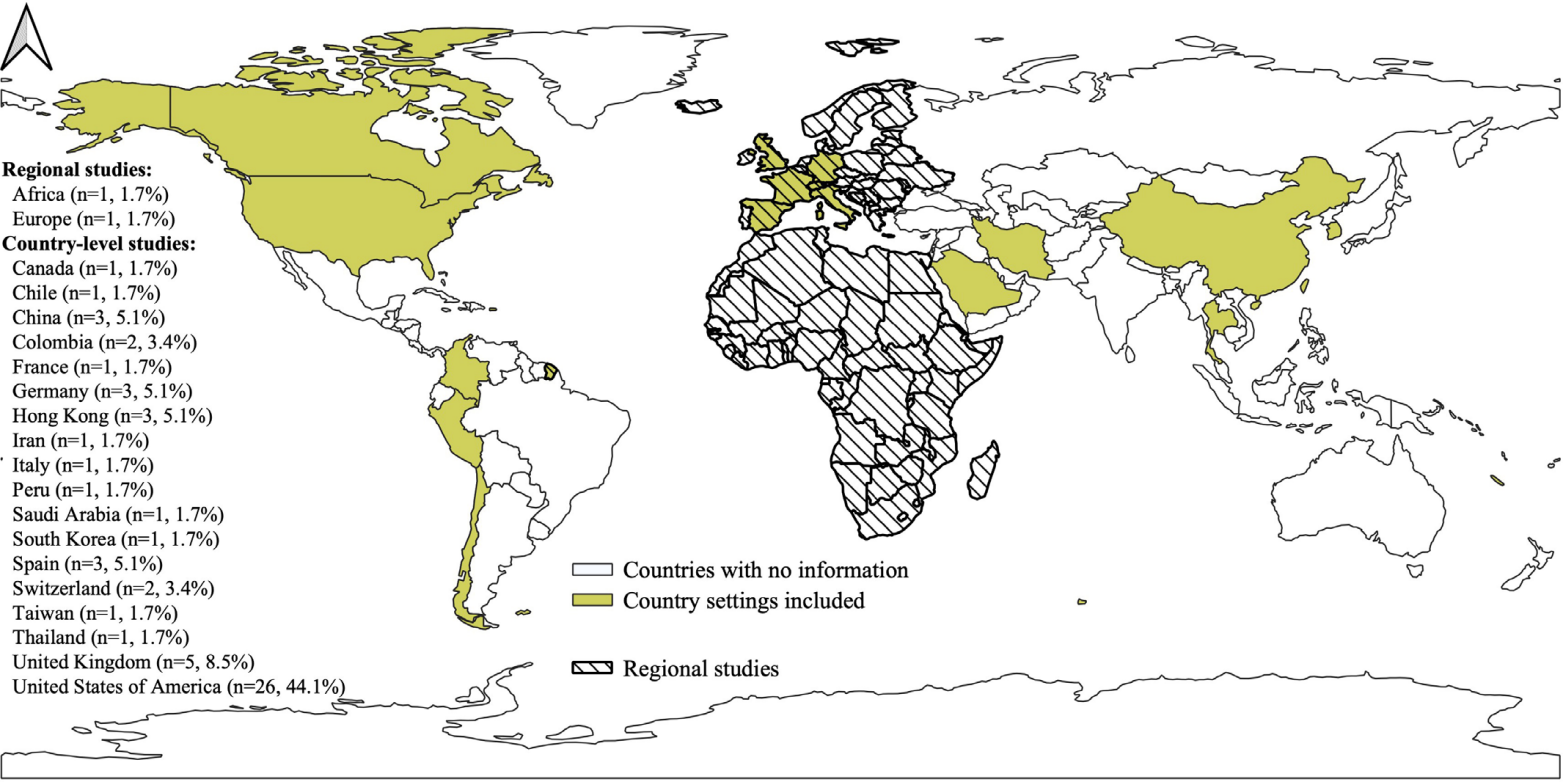
Geographical distribution of the included studies (N=59) Notes: Geographical Information System Open-Source Geospatial Foundation Project (QGIS) V.2022 was used for map visualisation.

Most reports on pharmaceutical interventions were focused on MRSA (20 of 32 studies, 63%). The remaining studies analysed carbapenem-resistant gram-negative pathogens contrasting ceftazidime avibactam versus colistin or alternative drug-based treatments. MRSA interventions were focused on comparing linezolid, or any relatively new drug (eg, daptomycin), with vancomycin, the established treatment. Studies on non-pharmaceutical interventions were wide-ranging but most explored surveillance or screening methods. Reports included improved surveillance and wide PCR or chromogenic-based surveillance and testing (n=11), multiple surveillance schemes including testing, decolonisation and/or isolation (n=8), infection control and hygiene including use of gowns and hand hygiene practices (n=3) and miscellaneous (n=5; eg, antibiotic stewardship, pre-emptive isolation, whole-genome sequencing). Generally, these interventions targeted MRSA (n=16, 59%), carbapenem-resistant Enterobacteriaceae (CRE) (n=4, 13%) and vancomycin-resistant Enterococci (VRE) (n=4), and compared the intervention’s effectiveness with current practice, which was typically the absence of the intervention. Most studies were conducted in high-income countries, mainly the USA (n=26, 44%; see figure 2). We found two regional studies; one using European data and the second in Africa. Decision analytical models were usually employed for the analyses (eg, decision trees, Markov and stochastic simulation models), often using a one-way sensitivity analysis. Time horizons and discount rates were reported inconsistently, and target populations usually consisted of all hospital patients and patients with pneumonia. See online supplemental tables SM6 and 7 for a full description of the studies’ characteristics.

### Unit costs of interventions

Online supplemental table SM8 provides a cost breakdown for pharmaceutical interventions. Economic costs varied based on factors such as drug components, dosage, length of hospital stay (LOS) and study scale. Bed-day expenses, associated with admissions to general wards and intensive care unit (ICU), constituted the largest portion of total economic costs (~50%–90%). Drugs represented about 10% of total costs (adjacent therapies, rehabilitation and diagnostic were costlier), with drugs like daptomycin and linezolid being notably more expensive, approximately 200% greater than vancomycin^24 25^ (online supplemental table SM8). For instance, Niederman *et al* reported the cost of intravenous linezolid (600 mg) as $107 per dose, while vancomycin costed $5.8 for 1 g intravenous administration.^26^

Online supplemental table SM9 shows an itemised breakdown of the non-pharmaceutical interventions’ unit costs. Hospitalisation and additional costs were the highest cost component. Test or intervention unit costs varied widely, ranging from $1 per patient (eg, use of gown or gloves^27^) to as high as $108 for genome sequencing,^28^ $103 for decolonisation,^29^ $598 for isolation^30^ and $652 for infection control bundles^31^ per patient. The lowest costs among non-pharmaceutical interventions were also those involving screening or surveillance, due to their being single-step procedures incurring no overhead or operating costs (eg, PCRs, chromogenic agar or electronic registry).

### Cost-effectiveness and outcomes

Online supplemental Table SM6 displays studies’ strategies and cost-effectiveness (eg, ICERs) of the pharmaceutical (I) and non-pharmaceutical (II) interventions.

#### Pharmaceutical interventions

##### Linezolid versus vancomycin

For patients with complicated skin and skin structure infections (cSSSI), linezolid consistently emerged as a cost-effective and dominant strategy compared with vancomycin (online supplemental table SM6, panel I).^24 32–35^ For instance, McKinnon *et al* 32 reported a mean cost of $7077 (SD=$5752) for linezolid versus $8709 (SD=$7307) for vancomycin treatment among patients with cSSSI reporting MRSA infections, with a mean cost difference of $2756 (p value=0.041) due a 2.5 days longer LOS for vancomycin-treated patients. Bounthavong *et al.*,^34^ De Cock *et al* 33 and Schürmann *et al* 35 estimated lower hospitalisation costs for linezolid (incremental costs were −$7791, −$1827 and −$1749, respectively) along with higher cure rates (incremental cure rates for first-line MRSA were 13%, 10% and 10%, respectively), compared with vancomycin in patients with cSSSI. Differences were explained by reduced LOS and improved treatment failures due to linezolid oral formulation compared with intravenous vancomycin therapy.

In studies focusing on nosocomial pneumonia,^25 26 36–43^ linezolid showed a dominant ICER or ICER ranging from $5726 to $84 823 per death averted or life saved, and between $3179 and $21 488 per cure or treatment success among MRSA-infected patients, compared with vancomycin (online supplemental table SM6, section I). Variations in LOS and its associated economic costs across study settings accounted for differences in ICER. Daniel Mullins *et al* predicted an ICER of $5726 for linezolid per life saved, balancing the higher acquisition costs with enhanced survival rates.^36^ De Cock *et al* designed a decision–analytical model using clinical trial data that again favoured linezolid over vancomycin with greater clinical cure (+8.7%) and survival (+13.2%) rates at an additional incremental cost of $420 per treatment cycle.^37^ However, Collins *et al* 25 reported a higher ICER per life saved ($84 823) due to limited variation in incremental mortality (≈1%) between linezolid and vancomycin.

**Figure 3:**
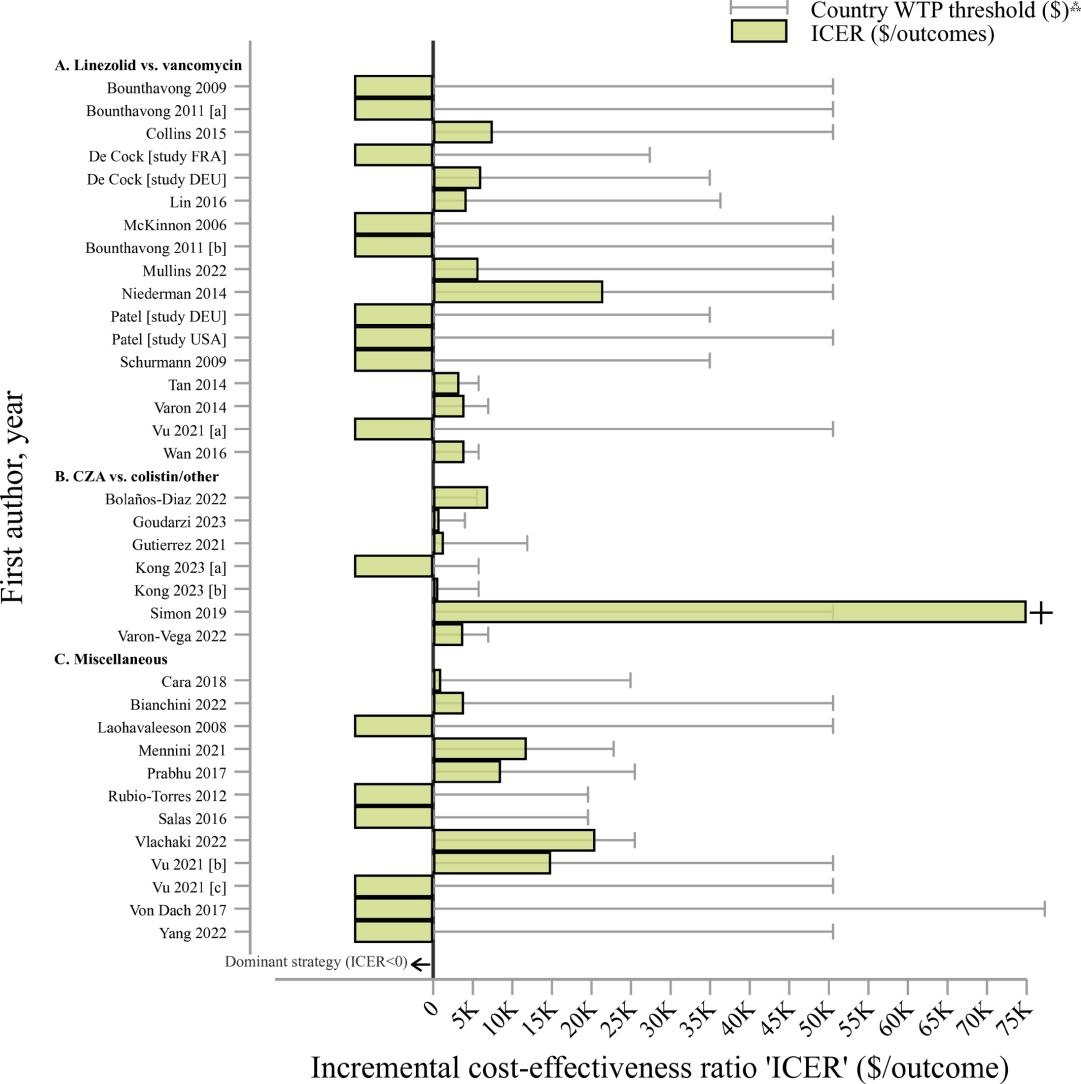
Incremental cost-effectiveness ratios and willingness-to-pay country thresholds among pharmaceutical interventions (in 2022 US dollars, ‘$’), by study†. Notes: †Studies with letters in brackets (eg, (a)) indicate different strategy evaluations, detailed in online supplemental table SM6 under the strategy column. K=thousands or 1000 units. Interpretation of the incremental cost-effectiveness ratio ‘ICER‘ should be taken with caution as outcomes (eg, deaths averted, cured patients, quality-adjusted life years ‘QALYs’) used to calculate ICERs varied from study to study. Online supplemental table SM6 contains detailed information by study and outcomes used. ^⁂^WTP thresholds were extracted from country estimates provided by Woods *et al* 22 and adjusted to 2022 US dollars. A dominant strategy means that interventions are more effective and less costly (ICER<0). We excluded ICER per life saved from Collins *et al* 25 and only ICER$ per QALY was included (ICER per life saved was far beyond the WTP threshold for this study, see online supplemental table SM6). + ICERs were capped at US$75 000 but values are higher (see online supplemental table SM6). CZA, ceftazidime avibactam; ‘vs’, versus; WTP, willingness-to-pay.

Figure 3A shows that the linezolid strategy is beneficial compared with vancomycin at country-specific WTP thresholds (ICER<WTP).

##### *Ceftazidime avibactam* versus colistin or other drugs

Six studies evaluated the use of ceftazidime avibactam (CZA) versus colistin or other drugs (online supplemental table SM6).^44–49^ ICERs ranged between $693 and $113 423 per QALY gained. Goudarzi *et al* 45 and Simon *et al* 47 calculated ICERs equal to $798 and $113 423 per QALY gained among patients infected with CRE, respectively, comparing CZA versus colistin therapy. Incremental QALYs were similar (≈0.5) in both studies, but costs differed. In Goudarzi *et al*, CZA therapy costs were 1.5-times greater for CZA compared with colistin according to Iran health system tariffs. Simon *et al* employed a healthcare system perspective in the USA, estimating four times greater daily therapy costs for CZA compared with colistin after accounting for LOS, which increased the ICER. In comparison to colistin+meropenem, Gutiérrez and Fandiño^48^ and Varón-Vega *et al* 49 reported ICERs of $1340 and $3797 per QALY gained for CZA, respectively. This difference is attributed to CZA showing increased incremental QALYs (+2.3 and +1.8, respectively), while incremental costs were similar ($3151 and $2886, respectively). The slight variation in additional concomitant treatments reported (amikacin+fosfomycin and tigecycline+fosfomycin) played a minor role.

Four studies presented an ICER below the WTP threshold (figure 3B), except Bolaños-Diaz *et al* 44 and Simon *et al.* 47

##### Miscellaneous: other combination drug comparison types

Laohavaleeson *et al* 50 found an estimated 0.5-day shorter LOS and savings of $478 favouring telavancin (dominant strategy compared with vancomycin) among MRSA patients, regardless of sensitivity analyses on MRSA drug acquisition costs. Favourable results were shown for IMI/REL (imipenem/cilastatin/relebactam) compared with CMS+IMI (colistin plus imipenem) usage for gram-negative infections (+3.7 QALYs and lower mortality rates; 15.2% compared with 39%). However, the clinical response rate was limited among the IMI/REL group.^51^ Additionally, treating patients with complicated intra-abdominal infections following ceftolozane/tazobactam+metronidazole was found to be cost-effective (ICER=$8551 per QALY gained), compared with piperacillin/tazobactam.^52^ Mennini *et al* 53 and Vlachaki *et al* 54 assessed meropenem-vaborbactam versus the best available treatment for CRE patients, revealing ICERs of $11 813 and $20 486 per QALY, respectively. The disparity arises from three times higher drug costs for meropenem-vaborbactam compared with the best available therapy in the UK,^54^ while in the Italy-based study,^53^ it was only 1.5 times higher. Furthermore, the UK-based study attributed higher costs to long-term care tariffs associated with increased survivability among meropenem-vaborbactam.

All miscellaneous interventions presented ICERs below country-specific WTP thresholds (figure 3C).

#### Non-pharmaceutical interventions

##### Testing schemes: chromogenic-based agar or PCR

**Figure 4:**
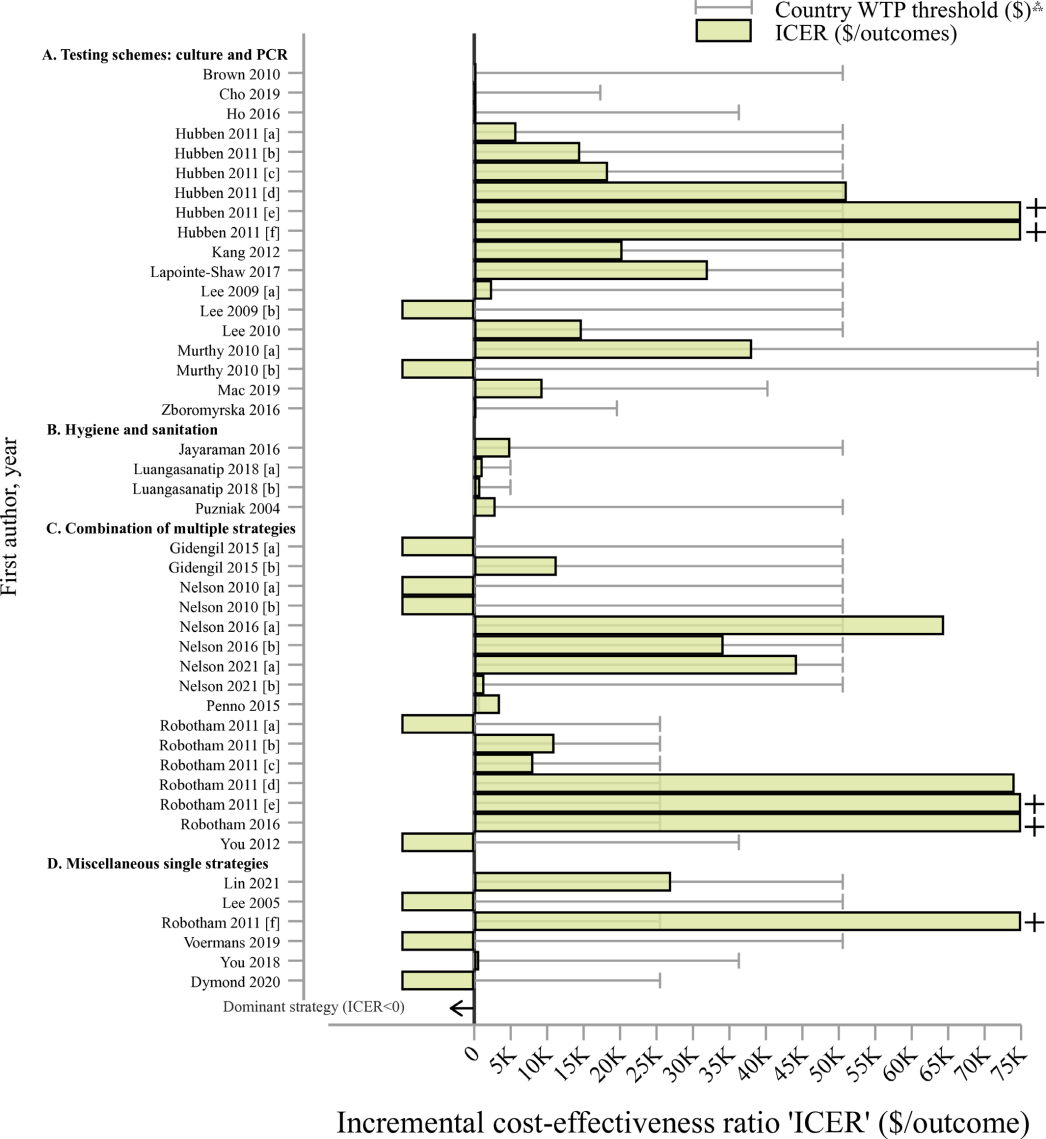
Incremental cost-effectiveness ratios and willingness-to-pay country thresholds among non-pharmaceutical interventions (in 2022 US dollars, ‘$’), by study†. Notes: †Studies with letters in brackets (eg, (a)) indicate different strategy evaluations, detailed in online supplemental table SM6 under the strategy column. K=thousands or 1000 units. Interpretation of the incremental cost-effectiveness ratio ‘ICER’ should be taken with caution as outcomes (eg, deaths averted, cured patients, quality-adjusted life years ‘QALYs’) used to calculate ICERs varied from study to study. Online supplemental table SM6 contains detailed information by study and outcomes used. ^⁂^WTP thresholds were extracted from country estimates provided by Woods *et al* 22 and adjusted to 2022 US dollars. A dominant strategy means that interventions is more effective and less costly (ICER<0). + ICERs were capped at US$75 000 but values are higher (see online supplemental table SM6). PCR, PCR chain reaction; ‘vs’, versus; WTP, willingness-to-pay.

Rapid PCR testing for MRSA detection compared with standard hospital treatments was found to be cost-effective (ICER=$55 and $39 per life-year saved in Europe and the USA, respectively^55^), with ICER=$20 401 per hospital-acquired MRSA case detected in the USA,^27^ ICER=$38 911 per MRSA infection averted in Switzerland^56^ and ICER=$243 per life year saved in Spain.^57^ Single-culture of an anterior nares specimen for universal screening of MRSA patients resulted in an ICER of $14 766 per QALY gained, compared with a ‘change nothing’ scenario, producing better MRSA control and lower losses attributed to hospital bed-day costs.^58^ One study showed that screening for carbapenemase-producing Enterobacteriaceae was cost-saving (ICER=$32 049 per QALY gained) at prevalence levels above 0.3% or if one additional patient were exposed for every infected patient (ie, highly dependent on local transmission settings).^59^ Similarly, active PCR among CRE patients, compared with do nothing, was cost-effective at $100 per QALY gained in surgical ICU patients in Hong Kong^60^ due to cheaper PCR unit costs compared with an inadequate empirical antibiotic treatment for CRE. Hubben *et al* 61 found selective chromogenic-based agar cost-effective for MRSA detection compared with taking no action (ICER= $5787–$14 538, with 622 infections averted in a moderate MRSA prevalence scenario). Selective PCR was also cost-effective versus chromogenic agar (ICER= $18 349–$51 095). However, universal screening was not cost-effective, as it incurred substantial costs for screening and isolation ($9.2 million incremental costs, with only 28 infections averted; ICER= $184 902–$328 448), surpassing the country WTP threshold (figure 4A).

##### Hygiene and sanitation

Interventions including proactive infection control, hand hygiene and gown usage were cost-effective at country WTP thresholds (figure 4B).^62–64^ For instance, Luangasanatip *et al* found that 20% compliance in healthcare hygiene protocol, versus 10%, was associated with reductions in MRSA bloodstream infections (BSIs) and ICERs of $1160 and $835 per QALY in paediatric and adult ICUs, respectively.^62^ Gown usage for 18 months was linked to 58 VRE cases averted in a hospital ICU in the USA (ICER=$2939 per case averted).^64^

##### Using a combination of multiple surveillance schemes and other methods

Combination schemes containing decolonisation, isolation, testing and surveillance were evaluated.^29 30 65–70^ Robotham *et al* combined screening, decolonisation and isolation techniques versus a do-nothing scenario.^29^ Universal PCR/chromogenic agar plus decolonisation with mupirocin was cost-effective finding up to $11 005 per QALY gained; however, most interventions involving patient isolation plus PCR for identification were costly due to infrastructure requirements (online supplemental table SM6, panel II; figure 4C). Universal decolonisation for ICU patients with MRSA infections emerged as a dominant strategy in the USA^68^ and in Hong Kong,^69^ leading to cost savings of $737 and reductions in infection and mortality rates by 0.9% and 0.2%, respectively. Similarly, Nelson *et al* 30 estimated that PCR screening and decolonisation (dominant strategy), had cost-savings of $14 433 and $47 762 and reduced 0.38 and 3.13 MRSA infections per 100 patients compared with PCR screening alone or do-nothing scenarios, respectively. However, in the same veteran hospital in the USA, more comprehensive strategies, comprising screening, contact precautions and infection control combined were more cost-effective, particularly in scenarios with high MRSA transmission rates rather than low transmission in subsequent periods (ICER= $13 904^66^ and $34 201^67^ per life years gained; as shown in online supplemental table SM6, panel II, and figure 4C). Last, real-time blood culturing and evidence-based antimicrobial consumption among ampicillin-resistant *Salmonella enterica* and *Streptococcus pneumoniae* infections were cost-effective in Africa (ICER=$3531 per life saved, averting 934 deaths per 100 000 patients), compared with generic antimicrobial management.^70^

Most of these strategies were cost-effective based on country WTP thresholds (figure 3C), but consideration of local costs was essential in scenarios with low MRSA prevalence and transmission.^65^

##### Miscellaneous single strategies

Interventions in this category included antibiotic stewardship, single surveillance schemes, test-guided decontamination and pre-emptive isolation.^28 31 71–73^ Voermans *et al* estimated that procalcitonin-led antibiotic stewardship reduced average expenses per patient, specifically, a 49% reduction from standard care for sepsis and 23% reduction for lower respiratory tract infections associated with ABR (cost savings of $29 197 and $4138 per each group).^72^ Active surveillance (current standards and screening of previously hospitalised) for patients with VRE was the most medically and economically beneficial, resulting in a $4 screening cost per patient admitted, lowering admission costs ($792) and improving survival rates.^71^ Whole genome sequencing as a surveillance alternative resulted in 14.3 additional QALYs gained among MRSA patients.^28^ The use of a state-wide electronic registry reduced CRE by 18.8 cases per year (95% CI=5.8 to 31.7) and by 6.3% (95% CI=2.0% to 10.6%; p value<0.05) compared with the ‘do nothing’ scenario (ICER=$27 000 per infection averted).^31^ Test-guided selective digestive decontamination among CRE patients in the ICU was cost-effective in reducing CRE (ICER=$688 per QALY, reduction of 0.2% and 0.3% in CRE cases and mortality, respectively).^73^ Most strategies were cost-effective according to country-specific WTP thresholds (figure 4D), except for Robotham *et al*’s study on universal pre-emptive isolation in the UK’s hospital ICU for high MRSA risk patients,^29^ which reported substantial hospital costs due to necessary infrastructure investments.

### Quality of reporting and risk of bias

A substantial proportion of the pharmaceutical (25%) and non-pharmaceutical studies (33%) failed to report important costs and their potential consequences (online supplemental table SM10). The type of costing methodology was dissimilar in studies, resulting in costs for drug acquisition reported, for instance, in cost per day, patient or dose. Discounting varied among studies in magnitude and usage (61% failed to report discounting online supplemental table SM10). Despite most studies achieving average high-quality scores of 8.2 and 8.0 out of 10 for pharmaceutical and non-pharmaceutical interventions,^74^ time frames and years of economic evaluation were not always reported.

## Discussion

We identified 59 studies investigating the cost-effectiveness of pharmaceutical or non-pharmaceutical interventions reducing ABR among WHO’s global priority pathogen list in hospital settings.^18^ We flag the reduced data among critical pathogens, such as *Acinetobacter baumannii* and *Pseudomonas aeruginosa*, and the scarcity of standardised cost-effectiveness methods, ingredient costs and limited data from low-income and middle-income countries indicated the need for more consistent approaches in the future.

More studies found that, compared with vancomycin, linezolid was more effective and less costly for the treatment of MRSA infections. Despite pharmaceutical costs being a highly predictable line item in hospital budgets (eg, diagnostic tests, treatment), LOS often constitutes a higher proportion of the cost for hospital stay and should be considered in cost-effectiveness analyses and decisions related to formulary and drug reimbursement. For example, Kauf *et al* reported that drug costs drove 6.4% of the total inpatient cost compared with LOS accounting for 85.9% of total inpatient cost for patients with cSSSI.^75^ Treatment resulting in expedited infection resolution will likely be more cost-effective even when drug costs are much higher. This is also seen with linezolid compared with vancomycin. Vancomycin can be taken orally (as opposed to intravenously) meaning that patients can be discharged earlier, potentially offsetting higher drug acquisition costs.^36^ De Cock *et al* noted that in a scenario analysis between linezolid and vancomycin, when the most conservative treatment durations were applied rather than those estimated by the physician panel, linezolid was dominant over vancomycin based on the shorter LOS.^33^

The appropriateness of initial antibiotic therapy and the possibility of switching treatments during hospitalisation also play crucial roles, by affecting length of hospital stay and treatment outcome. One key question is whether being on vancomycin during hospitalisation and switching to linezolid for outpatient care is cost-saving.^36^ De Cock *et al* suggest that most patients are cured after treatment with two lines of antibiotic therapy.^37^ Empirical therapy with linezolid was considered most cost-effective in unconfirmed MRSA patients, as LOS for unconfirmed patients is lower.^33^

A recent meta-analysis indicates that ceftazidime-avibactam offers advantages over colistin, including lower mortality rates, improved clinical cure rates and reduced kidney deterioration in CRE infections.^76^ Comparing ceftazidime-avibactam to colistin plus meropenem revealed high efficacy and lower nephrotoxicity in CRE patients in Chile^48^ and Colombia^49^ (ICER=$1340 and $3797 per QALY gained, both falling below the country’s WTP thresholds). This finding holds relevance for a region where the kidney disease burden is substantial.^77^ Moreover, considering the complex dosing requirements and close monitoring associated with colistin plus meropenem, along with the region’s higher prevalence of carbapenemase-producing Enterobacterales^78 79^ and antibiotic-resistant gram-negative pathogens,^80^ the potential for expanded treatment coverage is substantial.

Non-pharmaceutical interventions were generally less cost-effective than pharmaceutical interventions. For instance, one of the most expensive non-pharmaceutical interventions was a mandatory full National Health Service-level screening programme modelled by Robotham and colleagues.^65^ Other infrastructure-demanding interventions, such as whole genome sequencing (WGS), were only cost-effective if applied at a specific UK tertiary research hospital where MRSA prevalence was significant and sequencing infrastructure already existed.^28^ Although the effectiveness of WGS surveillance is highly dependent on infrastructure, the study’s modelling estimate found that WGS was not sensitive to simulated reduced efficacy in colonisation/mortality reduction.^28^ Nevertheless, the limited evidence renders universal screening strategies for reducing MRSA inconclusive.^81^ Literature on MRSA demonstrates the limited capacity to account for confounding and temporal trends when assessing the burden of disease and resource utilisation associated with MRSA screening.

Costs associated with the required professional training often lead to the perception that antimicrobial stewardship is not cost-effective. However, there might be unaccounted outcomes and positive spillover effects not captured by economic evaluations. Although not specifically targeting ABR, Scheetz, *et al* 82 presented an ICER of $3219 per QALY gained in antimicrobial stewardship programmes attributed to substantial fixed operating costs required to maintain the stewardship team and the reduction in patient inflow. Antimicrobial stewardship proves more economically efficient in larger hospitals with higher inpatient volume, presenting increased risks and expanded economic returns of scale, specifically for persuasive and structural programmes.^9^ Notwithstanding, some studies have shown mixed results, with increased consumption of antibiotics not targeted or restricted by the antimicrobial stewardship programme leading to higher global ABR rates and worsening patient outcomes.^83^ Decreased resistance may not be expected if antimicrobial stewardships only target certain antibiotics. LOS and mortality could be affected beyond antibiotic control, changes in preintervention and post-intervention populations, including existing comorbidities and disease severity, might lead to poorer health outcomes despite the stewardship programme.^83^ Comprehensive antimicrobial stewardship programmes, including physiological monitoring, therapy review and antibiotic restrictions are essential to avoid ABR and associated disease burden.

Procalcitonin (PCT) has demonstrated the ability to increase specificity and sensitivity for different bacterial infections at the point of care, even in the earliest phases of inflammation. PCT has been shown to reduce LOS and improve the appropriateness of antibiotic treatment at low costs compared with no-PCT.^72 84–86^ Similar to a study in Europe avoiding antibiotic days in European settings,^85^ we found support for PCT-guided healthcare in the USA, contributing to halving sepsis with cost-savings of $29 197 compared with costs for standard care.^72^ These results are mainly driven by the associated reduction in ICU-admitted patients, which results in shorter antibiotic treatment and exposure time. These findings are corroborated by studies by Mewes *et al,* Harrison and Collins and Huang *et al*, showing PCT to be a cost-saving strategy in hospitalised patients with lower respiratory tract infections or suspected sepsis,^87–89^ although not specifically targeting ABR pathogens. Furthermore, a recent study suggests that these interventions among emergency departments in low-resource settings are feasible if PCT is applied simultaneously with C-reactive protein through a fluorescence reader-based duplex lateral flow assay.^90^ This has direct implications for applications in low-income and middle-income countries for rapid and accurate viral and bacterial infection differentiation, with an estimated rounded cost per patient below $70.^90^

Reducing the time interval between a positive test for MRSA and the implementation of appropriate infection control measures during hospitalisation is achievable using diagnostic technologies such as PCR.^91^ PCR assays were cost-effective in Europe and the UK, with the lowest ICER values per life-saved, ranging from $1100 to $1200, compared with standard treatment.^55^ Although the costs are low, PCR is only feasible as an intervention when the hospital has appropriate facilities and when the additional delay incurred poses little-to-no threat to patient well-being. PCR-based interventions may only be cost-effective in highly endemic settings where targeted screening is likely to detect a large number of MRSA cases.^27^ Despite potential drawbacks, studies have shown that PCR may prevent adverse events and toxicity due to treating patients empirically,^92^ reducing LOS and economic costs.^93 94^

### Limitations

Our review has highlighted important deficiencies in the health economics literature pertaining to pharmaceutical and non-pharmaceutical interventions aimed at reducing, monitoring and controlling ABR levels, particularly concerning critical or high-priority bacteria. We included literature from three major search engines, potentially overlooking publications in interdisciplinary journals and grey literature like government reports, particularly from low-income and middle-income countries. Our primary sources were PubMed, which comprehensively indexes biomedical and life sciences literature, including health economics; Embase, which specialises in biomedical and pharmacological content, with a specific emphasis on drug and pharmaceutical research; and EconLit, which is dedicated to economics. Second, we found significant heterogeneity in the costs and effectiveness units reported across studies, which may have been affected by the lack of standardisation in analysis, illustrated by the scarcity of cost-utility analyses considering the difficulty of measuring quality of life for acute events. Therefore, comparing results was challenging given the range of resistant bacterial types, intervention types, populations studied and the lack of consistency in study design. Our study focused on the health systems perspective to report unit costs and cost-effectiveness, which fails to take account of a societal perspective. However, most studies did not report a specific perspective of analysis. Finally, many articles failed to report discounting and a risk scenario for the associated consequences. This may be explained because due to the short time horizons used, often under a year and mostly under a month, which may not capture all relevant costs and benefits of the interventions. While we used Woods *et al’s* cost-effectiveness or WTP thresholds,^22^ some literature suggests wider thresholds, such as $100 000 or $150 000 per QALY, as more appropriate for evaluating interventions in the USA. This variation might impact the generalisability of our results.^95 96^ It is relevant to recall that cost-effectiveness thresholds are contingent on the locally-relevant WTP thresholds.

## Conclusion

Most economic evaluations on ABR interventions have focused on MRSA, revealing a significant gap for other priority pathogens. Even when available, most studies lack a comprehensive economic analysis, even though such analysis would require readily available components such as intervention costs, bed-day expenses and patient outcomes, such as LOS or ICU admission. Data on bed-day expenses for primary, secondary and tertiary hospitals are freely available for most countries from the WHO-CHOICE.^97^ This is important because, as Nathwani *et al* 83 showed, more effective antimicrobial control does not necessarily translate into improved cost-effectiveness due to population heterogeneity and decisions in resource allocation. Many studies were based on non-randomised designs that did not adequately account for potential confounders and antimicrobial regulations or guidelines (eg, stewardship programmes could reduce antibiotic consumption of a targeted component while increasing others). This issue could be rectified by strengthening intervention designs through a priori examination of biases and ensuring consistency. We have synthesised evidence supporting pharmacological and non-pharmacological interventions from the limited available scientific literature using economic analysis. Still, for many interventions, hospital-level considerations (eg, laboratory capacity, the prevalence of resistance in the local community, therapy review and population features) need to be considered to optimise healthcare expenditure and address the costs of inaction. We recommend future economic evaluations consider the Consolidated Health Economic Evaluation Reporting Standards checklist^98^ using the healthcare sector and societal perspectives simultaneously as benchmarks^99^ and for consistency across studies.

## Data Availability

All data relevant to the study are included in the article or uploaded as supplementary information.
