# Supplementary material for: Costs-effectiveness and cost components of pharmaceutical and non-pharmaceutical interventions affecting antibiotic resistance outcomes in hospital patients: a systematic literature review
Source: BMJ Glob Health. 2024 Feb 29;9(2):e013205. doi: 10.1136/bmjgh-2023-013205 (PMC10910705; doi:10.1136/bmjgh-2023-013205)
Supplement: Supplementary data [file bmjgh-2023-013205supp001.pdf]

SUPPLEMENTARY MATERIAL

Costs-effectiveness and cost components of pharmaceutical and non-pharmaceutical interventions affecting antibiotic resistance outcomes in hospital patients: A systematic literature review

Kasim Allel[*], María José Hernández-Leal, Nichola Naylor, Eduardo A. Undurraga, Gerard Joseph Abou Jaoude, Priyanka Bhandari, Ellen Flanagan, Hassan Haghparast-Bidgoli, Koen B. Pouwels, Laith Yakob

Table of Contents

Table SM1: Search strategy 2

Table SM2: Study inclusion and exclusion criteria 3

Table SM3: WHO global priority pathogens list of antibiotic-resistant bacteria 4

Table SM4: Final literature search strategy (search codes) in three search engines (12th of December 2023) 5

Table SM5: Drummond's checklist for assessing economic evaluations 7

Table SM6. Reported cost-effectiveness per study (in 2022 USDs) and intervention type (pharmaceutical and non-pharmaceutical, N=59 studies) 9

Table SM7. Characteristics of the included studies (n=59) 14

Table SM8. Unit costs per study for pharmacological interventions (in 2022 USDs) 17

Table SM9. Unit costs per study for non-pharmaceutical interventions (in 2022 USDs) 29

Table SM10: Quality appraisal using Drummond's checklist 37

Table SM11: Prisma Checklist[65] 39

References 43

[†] Supplementary file containing cost ingredients per study is located in https://bit.ly/SR_amrCEingredients.

[*] **Corresponding author**. Institute for Global Health, 3rd floor, Institute of Child Health, 30 Guilford Street, London WC1N 1EH, email: k.allel@ucl.ac.uk

Allel K, *et al. BMJ Glob Health* 2024; 9:e013205. doi: 10.1136/bmjgh-2023-013205

Table SM1: Search strategy

**Research question**	**What is the economic impact (cost-effectiveness) of pharmaceutical and non-pharmaceutical interventions for reducing AMR levels among critical pathogens within hospital inpatients?**		
**Keywords**	Economic evaluation	Population	Antimicrobial
**Search terms**	Economics<br>Costs<br>Cost Analysis<br>Fees and Charges<br>Budgets<br>Pharmacoeconomic<br>Expenditure<br>Finance	Hospital<br>Patient<br>Inpatient	Antimicrobial<br>Microbial<br>Antibiotic<br>+<br><br>Resistance

Table SM2: Study inclusion and exclusion criteria

Criteria	Inclusion Criteria	Exclusion Criteria
**Population**	Hospitalised patients, no age restrictions	Patients outside hospital
**Geography**	All countries	None
**Period**	Until December 2023	After December 2023
**Setting**	Inpatients care setting, hospital infections, nosocomial infections (infections occurring within the hospital)	Nursing home, long-term care studies, community settings.
**Interventions**	Pharmaceutical or non-pharmaceutical interventions targeting infections from the WHO critical and high-priority AMR bacterial pathogens only	All other interventions or pathogens.
**Outcomes**	Studies must have at least an incremental cost-effectiveness measure, e.g., dollars per QALY gained, however, other measures were included ,e.g. cost per patient cured	All other outcomes (non-incremental cost per gain in hospital outcomes).
**Publication language**	All languages	None
**Publication Type**	Peer-reviewed articles	Conference proceedings, case reports, grey literature, magazine entries, protocols, literature reviews, commentaries, and abstracts
**Study design**	Cost-effectiveness analyses, cost-utility analyses, cost-benefit analyses, piggyback economic evaluation alongside RCTs, case reports	All other study designs (e.g., literature review,; systematic reviews; meta-analyses not using primary data)

Notes: QALY: quality-adjusted life year. RCT= randomised controlled trial.

Table SM3: WHO global priority pathogens list of antibiotic-resistant bacteria

Priority 1: CRITICAL
• *Acinetobacter baumannii*, carbapenem-resistant<br>• *Pseudomonas aeruginosa*, carbapenem-resistant<br>• *Enterobacteriaceae*, carbapenem-resistant, ESBL-producing
**Priority 2: HIGH**
• *Enterococcus faecium*, vancomycin-resistant<br>• *Staphylococcus aureus*, methicillin-resistant, vancomycin-intermediate, and resistant<br>• *Helicobacter pylori*, clarithromycin-resistant<br>• *Campylobacter spp.*, fluoroquinolone-resistant<br>• *Salmonellae*, fluoroquinolone-resistant<br>• *Neisseria gonorrhoeae*, cephalosporin-resistant, fluoroquinolone-resistant
**Priority 3: MEDIUM**
• *Streptococcus pneumoniae*, penicillin-non-susceptible<br>• *Haemophilus influenzae*, ampicillin-resistant<br>• *Shigella spp.*, fluoroquinolone-resistant

Notes: Adapted from the World Health Organization ‘WHO’ priority pathogen report[1].

Table SM4: Final literature search strategy (search codes) in three search engines (12th of December 2023)

I. PubMed

('Economic' OR 'Budget' OR 'cost' OR 'cost analysis' OR 'pharmacoeconomic' OR 'pharmaco-economic' OR 'economic evaluation' OR 'economic analysis' OR 'economic modelling' OR 'cost utility' OR 'cost minimi*' OR 'cost' OR 'cost saving' OR 'cost-saving' OR 'cost allocation' OR 'expenditure' OR 'expense' OR 'financ*' OR 'healthcare cost' OR 'unit cost' OR 'money' OR 'monetary' OR 'cost-effectiv*' OR 'cost-benefit') AND ('Drug resistance' OR 'antimicrobial drug resistan*' OR 'drug resistan*' OR 'antibiotic resistan*' OR 'antimicrobial resistan*' OR 'multi-drug resistan*' OR 'drug-resistance' OR 'carbapenem-resistant Escherichia coli' OR 'carbapenem-resistant Klebsiella pneumoniae' OR 'cephalosporin-resistant Escherichia coli' OR 'cephalosporin-resistant Klebsiella pneumoniae' OR 'carbapenem-resistant Enterobacteral*' OR 'carbapenem-resistant Enterobacteriaceae' OR 'cephalosporin-resistant Enterobacteral*' OR 'cephalosporin-resistant Enterobacteriaceae' OR 'Penicillin-resistant Streptococcus pneumoniae' OR 'vancomycin-resistant Staphylococcus aureus' OR 'methicillin-resistant Staphylococcus aureus' OR 'carbapenem-resistant Pseudomonas aeruginosa' OR 'carbapenem-resistant Acinetobacter baumanii' OR 'vancomycin-resistant Enterococcus' OR 'vancomycin-resistant Enterococcus faecium' OR 'clarithromycin-resistant Helicobacter pylori' OR 'fluoroquinolone-resistant Campylobacter' OR 'fluoroquinolone-resistant Salmonella' OR 'fluoroquinolone-resistant Neisseria gonorrhoeae' OR 'cephalosporin-resistant Neisseria gonorrhoeae' OR 'fluoroquinolone-resistant Shigella' OR 'ampicillin-resistant Haemophilus influenzae') AND ('hospital' OR 'inpatient' OR 'patient' OR 'healthcare' OR 'ICU' OR 'intensive care unit' OR 'ward' OR 'clinic' OR 'medical' OR 'nursing') NOT ('HIV' OR 'Tuberculosis' OR 'TB' OR 'virus' OR 'fungus' OR 'fungal' OR 'conference' OR 'letter to the editor')

II. EMBASE

((('Economic' or 'Budget' or 'cost' or 'cost analysis' or 'pharmacoeconomic' or 'pharmaco-economic' or 'economic evaluation' or 'economic analysis' or 'economic modelling' or 'cost utility' or 'cost minimi*' or 'cost' or 'cost saving' or 'cost-saving' or 'cost allocation' or 'expenditure' or 'expense' or 'financ*' or 'healthcare cost' or 'unit cost' or 'money' or 'monetary' or 'cost-effectiv*' or 'cost-benefit') and ('Drug resistance' or 'antimicrobial drug resistan*' or 'drug resistan*' or 'antibiotic resistan*' or 'antimicrobial resistan*' or 'multi-drug resistan*' or 'drug-resistance' or 'carbapenem-resistant Escherichia coli' or 'carbapenem-resistant Klebsiella pneumoniae' or 'cephalosporin-resistant Escherichia coli' or 'cephalosporin-resistant Klebsiella pneumoniae' or 'carbapenem-resistant Enterobacteral*' or 'carbapenem-resistant Enterobacteriaceae' or 'cephalosporin-resistant Enterobacteral*' or 'cephalosporin-resistant Enterobacteriaceae' or 'Penicillin-resistant Streptococcus pneumoniae' or 'vancomycin-resistant Staphylococcus aureus' or 'methicillin-resistant Staphylococcus aureus' or 'carbapenem-resistant Pseudomonas aeruginosa' or 'carbapenem-resistant Acinetobacter baumanii' or 'vancomycin-resistant Enterococcus' or 'vancomycin-resistant Enterococcus faecium' or 'clarithromycin-resistant Helicobacter pylori' or 'fluoroquinolone-resistant Campylobacter' or 'fluoroquinolone-resistant Salmonella' or 'fluoroquinolone-resistant Neisseria gonorrhoeae' or 'cephalosporin-resistant Neisseria gonorrhoeae' or 'fluoroquinolone-resistant Shigella' or 'ampicillin-resistant Haemophilus influenzae') and ('hospital' or 'inpatient' or 'patient' or 'healthcare' or 'ICU' or 'intensive care unit' or 'ward' or 'clinic' or 'medical' or 'nursing')) not ('HIV' or 'Tuberculosis' or 'TB' or 'virus' or 'fungus' or 'fungal' or 'conference' or 'letter to the editor')).mp. [mp=title, abstract, heading word, drug trade name, original title, device manufacturer, drug manufacturer, device trade name, keyword heading word, floating subheading word, candidate term word]

III. Econlit

('Economic Development' OR 'Model' OR 'Economic' OR 'fee' OR 'charge' OR 'Budget' OR 'cost' OR 'cost analysis' OR 'pharmacoeconomic' OR 'pharmaco-economic' OR 'pricing' OR 'economic evaluation' OR 'economic analysis' OR 'economic modelling' OR 'cost utility' OR 'cost minimi*' OR 'cost' OR 'cost saving' OR 'cost allocation' OR 'expenditure' OR 'expense' OR 'finance*' OR 'healthcare cost' OR 'unit cost' OR 'money' OR 'monetary') AND ('Drug resistance' OR 'antimicrobial drug resistan*' OR 'drug resistan*' OR 'antibiotic resistan*' OR 'antimicrobial resistan*' OR 'multi-drug resistan*' OR 'drug-resistance' OR 'carbapenem-resistant Escherichia coli' OR 'carbapenem-resistant Klebsiella pneumoniae' OR 'cephalosporin-resistant Escherichia coli' OR 'cephalosporin-resistant Klebsiella pneumoniae' OR 'carbapenem-resistant Enterobacteral*' OR 'carbapenem-resistant Enterobacteriaceae' OR 'cephalosporin-resistant Enterobacteral*' OR 'cephalosporin-resistant Enterobacteriaceae' OR 'Penicillin-resistant Streptococcus pneumoniae' OR 'vancomycin-resistant Staphylococcus aureus' OR 'methicillin-resistant Staphylococcus aureus' OR 'carbapenem-resistant Pseudomonas aeruginosa' OR 'carbapenem-resistant Acinetobacter baumanii' OR 'vancomycin-resistant Enterococcus' OR 'vancomycin-resistant Enterococcus faecium' OR 'clarithromycin-resistant Helicobacter pylori' OR 'fluoroquinolone-resistant Campylobacter' OR 'fluoroquinolone-resistant Salmonella' OR 'fluoroquinolone-resistant Neisseria gonorrhoeae' OR 'cephalosporin-resistant Neisseria gonorrhoeae' OR 'fluoroquinolone-resistant Shigella' OR 'ampicillin-resistant Haemophilus influenzae') AND ('hospital' OR 'inpatient' OR 'patient' OR 'healthcare' OR 'ICU' OR 'intensive care unit' OR 'ward' OR 'clinic' OR 'medical' OR 'nursing')

Allel K, *et al. BMJ Glob Health* 2024; 9:e013205. doi: 10.1136/bmjgh-2023-013205

Table SM5: Drummond's checklist for assessing economic evaluations

1. Was a well-defined question posed in answerable form?

1.1. Did the study examine both costs and effects of the service(s) or programme(s)?
1.2. Did the study involve a comparison of alternatives?
1.3. Was a viewpoint for the analysis stated, and was the study placed in any particular decision-making context?

2. Was a comprehensive description of the competing alternatives given (i.e., can you tell who did what to whom, where, and how often)?

2.1. Were there any important alternatives omitted?
2.2. Was (should) a do-nothing alternative be considered?

3. Was the effectiveness of the programme or services established?

3.1. Was this done through a randomised, controlled clinical trial? If so, did the trial protocol reflect what would happen in regular practice?
3.2. Was effectiveness established through an overview of clinical studies?
3.3. Were observational data or assumptions used to establish effectiveness? If so, what are the potential biases in results?

4. Were all the important and relevant costs and consequences for each alternative identified?

4.1. Was the range wide enough for the research question at hand?
4.2. Did it cover all relevant viewpoints? (Possible viewpoints include the community or social viewpoint, and those of patients and third-party payers. Other viewpoints may also be relevant depending upon the particular analysis.)
4.3. Were the capital costs, as well as operating costs, included?

5. Were costs and consequences measured accurately in appropriate physical units (e.g., hours of nursing time, number of physician visits, lost work days, gained life years)?

5.1. Were any of the identified items omitted from measurement? If so, does this mean they carried no weight in the subsequent analysis?
5.2. Were there special circumstances (e.g., joint use of resources) that made measurement difficult? Were these circumstances handled appropriately?

6. Were the cost and consequences valued credibly?

6.1. Were the sources of all values clearly identified? (Possible sources include market values, patient or client preferences and views, policy-makers views, and health professionals' judgements)
6.2. Were market values employed for changes involving resources gained or depleted?
6.3. Where market values were absent (e.g. ,volunteer labour), or market values did not reflect actual values (such as clinic space donated at a reduced rate), were adjustments made to approximate market values?
6.4. Was the valuation of consequences appropriate for the question posed (i.e., has the appropriate type or types of analysis – cost-effectiveness, cost-benefit, cost-utility – been selected)?

7. Were costs and consequences adjusted for differential timing?

Allel K, *et al. BMJ Glob Health* 2024; 9:e013205. doi: 10.1136/bmjgh-2023-013205

7.1. Were costs and consequences that occur in the future 'discounted' to their present values?
7.2. Was there any justification given for the discount rate used?

8. Was an incremental analysis of costs and consequences of alternatives performed?

8.1. Were the additional (incremental) costs generated by one alternative over another compared to the additional effects, benefits, or utilities generated?

9. Was allowance made for uncertainty in the estimates of costs and consequences?

9.1. If data on costs and consequences were stochastic (randomly determined sequence of observations), were appropriate statistical analyses performed?
9.2. If a sensitivity analysis was employed, was justification provided for the range of values (or key study parameters)?
9.3. Were the study results sensitive to changes in the values (within the assumed range for sensitivity analysis, or within the confidence interval around the ratio of costs to consequences)?

10. Did the presentation and discussion of study results include all issues of concern to users?

10.1. Were the conclusions of the analysis based on some overall index or ratio of costs to consequences (e.g., cost-effectiveness ratio)? If so, was the index interpreted intelligently or in a mechanistic fashion?
10.2. Were the results compared with those of others who have investigated the same question? If so, were allowances made for potential differences in study methodology?
10.3. Did the study discuss the generalisability of the results to other settings and patient/client groups?
10.4. Did the study allude to, or take account of, other important factors in the choice or decision under consideration (e.g.,. distribution of costs and consequences, or relevant ethical issues)?
10.5. Did the study discuss implementation issues, such as the feasibility of adopting the 'preferred' programme given existing financial or other constraints, and whether any freed resources could be redeployed to other worthwhile programmes?

Notes: Drummond, et al. 2015 [2].

Table SM6. Reported cost-effectiveness per study (in 2022 USDs) and intervention type (pharmaceutical and non-pharmaceutical, N=59 studies)

First author	Year	Country	Pathogen	Hospital population	Strategy	ICER
**I. Pharmaceutical interventions** (N= 32 studies)						
Bianchini[3]	2022	USA	CRO	All	New beta-lactam beta-lactamase Inhibitor antibiotics‡ vs. colistin treatment	$3,900/QALY.
Bolaños-Diaz[4]	2022	PER	CRE	BSI and Pneumonia	Ceftazidime avibactam‡ vs. colistin-based treatment.	$6,947/QALY.
Bounthavong[5]	2009	USA	MRSA	cSSSI	Linezolid‡ vs. vancomycin treatment.	Dominant strategy ($/cure).
Bounthavong[6]	2011	USA	MRSA	cSSSI	[a] Linezolid‡ vs. vancomycin treatment.<br><br>[b] Linezolid‡ vs. daptomycin treatment.	Dominant strategy ($/treatment success).<br>Dominant strategy ($/treatment success).
Cara[7]	2018	KSA	MDR GN	Pneumonia	Low dose of colistin‡ vs. high dose of colistin treatment.	$1,006/nephrotoxicity avoided.
Collins[8]	2015	USA	MRSA	Pneumonia	Linezolid‡ vs. vancomycin treatment.	$7,527/QALY and $84,823/life saved
De Cock[9]	2009	FRA	MRSA	cSSSI	Linezolid‡ vs. vancomycin treatment.	Dominant strategy ($/cure & $/death averted).
De Cock[10]	2009	DEU	MRSA	Pneumonia	Linezolid‡ vs. vancomycin treatment.	$9,223/cure; $6,076/death averted; $345/LY.
Goudarzi[11]	2023	IRN	CRE	All	Ceftazidime avibactam‡ vs. colistin treatment.	$798/QALY.
Gutierrez[12]	2021	CHL	CRE	All	Ceftazidime/avibactam‡ vs. colistin+ meropenem treatment.	$1,340/QALY and $1,342/LY
Kong[13]	2023	CHN	CRE	BSI	[a] Ceftazidime-avibactam‡ vs. polymyxin B (PMB) monotherapy.<br><br>[b] Ceftazidime-avibactam‡ vs. PMB-based therapy.	Dominant strategy ($/QALY).<br><br>$639/QALY.
Laohavaleeson[14]	2008	USA	MRSA	cSSSI	Telavancin‡ vs. vancomycin treatment.	Dominant strategy ($/cure).
Lin[15]	2016	TWN	MRSA	Pneumonia	Linezolid‡ vs. vancomycin treatment.	$4,224/cure.
McKinnon[16]	2006	USA	MRSA	cSSSI	Linezolid‡ vs. vancomycin treatment.	Dominant strategy ($/% cure rate).
Mennini[17]	2021	ITA	CRE	All	Vaborem (meropenem-vaborbactam)‡ versus best available treatment.	$9,548/LY and $11,813/QALY.
Mullins[18]	2022	USA	MRSA	Pneumonia	Linezolid‡ vs. vancomycin treatment.	$5,726/life saved.
Niederman[19]	2014	USA	MRSA	Pneumonia	Linezolid‡ vs. vancomycin treatment.	$21,488/treatment success.
Patel[20]	2014	DEU	MRSA	Pneumonia	Linezolid‡ vs. vancomycin treatment.	Dominant strategy ($/treatment success).
Patel[21]	2014	USA	MRSA	Pneumonia	Linezolid‡ vs. vancomycin treatment.	Dominant strategy ($/treatment success).
Prabhu[22]	2017	GBR	ABR GN	IAI	Ceftolozane/tazobactam/ metronidazole‡ vs. piperacillin/tazobactam treatment.	$8,551/QALY.
Rubio-Terres[23]	2012	ESP	MRSA	All	Daptomycin‡ vs. vancomycin treatment.	Dominant strategy ($/cure).

Salas[24]	2016	ESP	MRSA	Post-surgery	Intense mupirocin treatment among MRSA colonised patients‡ vs. conventional mupirocin treatment.	$44,552/infection averted.
Schurmann[25]	2009	DEU	MRSA	cSSSI	Linezolid‡ vs. vancomycin treatment.	Dominant strategy ($/cure).
Simon[26]	2019	USA	CRE	BSI and Pneumonia	Ceftazidime-avibactam‡ vs. colistin-based treatment.	$113,423/QALY.
Tan[27]	2014	CHN	MRSA	Pneumonia	Linezolid‡ vs. vancomycin treatment calibrated to different cities.	Up to $3,312/ treatment success.
Varon[28]	2014	COL	MRSA	Pneumonia	Linezolid‡ vs. vancomycin treatment.	$3,179/cure.
Varon-Vega[29]	2022	COL	CRE	Pneumonia	Ceftazidime-avibactam‡ vs. colistin-meropenem treatment.	$3,797/QALY.
Vlachaki[30]	2022	GBR	CRE	All	Vaborem (meropenem-vaborbactam)‡ versus best available treatment.	$20,486/QALY.
Vu[31]	2021	USA	MRSA	BSI	[a] Linezolid‡ vs. vancomycin 4-weeks treatment.<br><br>[b] Daptomycin‡ vs. linezolid 4-weeks treatment.<br><br>[c] Linezolid‡ vs. ceftaroline/daptomycin 4-weeks treatment.	Dominant strategy ($/treatment failure avoided).<br>$14,881/treatment failure avoided.<br><br>Dominant strategy ($/treatment failure avoided).
Von Dach[32]	2017	CHE	MRSA	All	Trimethoprim-sulfamethoxazole + rifampicin‡ vs. linezolid.	Dominant strategy($/QALY).
Wan[33]	2016	CHN	MRSA	Pneumonia	Linezolid‡ vs. vancomycin treatment calibrated to different cities.	Up to $3,984/ treatment success.
Yang[34, 35]	2022	USA	CR-GN	All	Imipenem/cilastatin/relebactam‡ vs. colistin/imipenem treatment.	Dominant strategy ($/QALY).
**II. Non-pharmaceutical interventions** (N= 27 studies)						
Brown[36]	2010	EU & USA	MRSA	All	Rapid PCR testing‡ vs. empiric vancomycin treatment.	$55 (EU) and $39 (USA) /LY.
Cho[37]	2019	KOR	CLRHP	All	DPO-based multiplex PCR therapy‡ vs. conventional therapy.	$5/case eradicated.
Dymond[38]	2020	GBR	MRSA	All	Whole genome sequencing‡ vs. standard infection control.	Dominant strategy ($/QALY).
Gidengil[39]	2015	USA	MRSA	ICU	[a] Universal decolonisation‡ vs. standard infection control.<br><br>[b] Universal contact precautions + decolonisation‡ vs. universal decolonisation.	Dominant strategy ($/colonisation or death averted).<br><br>$3,102/ colonisation averted and $11,316/ infection averted.
Ho[40]	2016	HKG	CRE	Surgical ICU	Active surveillance (PCR) + isolation of CRE+‡ vs. no surveillance.	$100/QALY.
Hubben[41]	2011	USA	MRSA	All	[a][b] Selective chromogenic-based screening in high and medium prevalence settings‡ vs. do nothing.<br><br>[c][d] Selective PCR-based tests in high and medium prevalence settings‡ vs. selective chromogenic-based screening.<br><br>[e][f] Universal screening with PCR-based tests in high and medium prevalence settings‡ vs. selective PCR-based test	$5,787 and $14,538/ case averted, respectively.<br><br>$18,349 and $51,095 per case averted, respectively.<br><br>$184,902 and $328,448/ case

						averted, respectively.
Jayaraman[42]	2016	USA	MDR	ICU	Proactive infection control program (enhanced hand hygiene, cleaning wards, increased nurse-to-patient ratio, and replacement of all disposable supplies)‡ vs. standard of care.	$4,949/transmission averted.
Kang[43]	2012	USA	MRSA	All	PCR-universal screening surveillance‡ vs. no surveillance.	$20,401/detected case.
Lapointe-Shaw[44]	2017	USA	CRE	All	Universal screening surveillance (PCR/culture)‡ vs. no surveillance	$32,049/QALY.
Lee[45]	2005	USA	VRE	All	Screening utilising current standards plus those patients with hospitalisations in previous 2-years‡ vs. current standards.	Dominant strategy ($/death averted)
Lee[46]	2009	USA	MRSA	Surgery	[a] Universal preoperative screening (culture of a single anterior nares sample)‡ vs. do nothing at MRSA prevalence of 0.1 in a single location.	$2,452/QALY.
					[b] Universal preoperative screening (culture of a single anterior nares sample)‡ vs. doing nothing at MRSA prevalence >0.1 in a single location.	Dominant strategy ($/QALY).
Lee[47]	2010	USA	MRSA	All	Universal screening surveillance (culture of a single anterior nares sample)‡ vs. no surveillance.	$14,766/QALY.
Lin[48]	2021	USA	CRE	All	Screening surveillance schemes using electronic registry (state-wide and hospital records)‡ vs. doing nothing scenario.	$27,000/ infection averted
Luangasanatip[49]	2018	THA	MRSA	BSI, ICU	[a] Hand hygiene intervention to improve compliance at 20%, 30% and 40%‡ vs. hand hygiene compliance at 10% in paediatric ICU.	$1,160, $806, and $739/QALY.
					[b] Hand hygiene intervention to improve compliance at 20%, 30% and 40%‡ vs. hand hygiene compliance at 10% in adult ICU.	$835, $574, and $524/QALY.
Mac[50]	2019	CAN	VRE	General ward	Screening (swabs and culture) and isolation‡, compared to no screening or isolation.	$9,372/QALY.
Murthy[51]	2010	CHE	MRSA	Surgery	[a] PCR screening at admission‡ vs. no screening.	$38,111/infection avoided.
					[b] PCR screening at admission‡ vs. screening for risk factors + isolation.	Dominant strategy ($/infection avoided).
Nelson[52]	2010	USA	MRSA	All	[a] Active surveillance (PCR screening)+ decolonization‡ vs. active surveillance alone.	Dominant strategy ($/infections or deaths avoided).
					[b] Active surveillance (PCR screening) + decolonization‡ vs. no surveillance.	Dominant strategy ($/infections or deaths avoided).
Nelson[53]	2016	USA	MRSA	HAI	[a][b] 3-year hospital surveillance initiative including screening, contact precautions, improved hand hygiene and infection control‡ vs. no initiative.	Between $34,201 and $64,436/LY, subject to high and low transmission.

Nelson[54]	2021	USA	MRSA and VRE	HAI	[a][b] 3-year hospital surveillance initiative including screening, contact precautions, improved hand hygiene and infection control‡ vs. no initiative.	Between $13,904 and $44,270/LY, subject to high and low transmission
Penno[55]	2015	Africa	PRSP	BSI	Evidence-based antimicrobial surveillance using local data and blood cultures‡ vs. generic antimicrobial management	$3,531/life saved.
Puzniak[56]	2004	USA	VRE	ICU	Use of gown and gloves‡ vs. gloves alone on entry to patient rooms.	$2,939/case averted.
Robotham[57]	2011	GBR	MRSA	ICU	[a] Universal chromogenic agar screening and decolonisation with mupirocin‡ vs. do nothing.<br><br>[b] Universal PCR and decolonisation with mupirocin‡ vs. do nothing.<br><br>[c] Chromogenic agar screening for high-risk patients and isolation of MRSA+‡ vs. do nothing.<br><br>[d] PCR for high-risk patients and isolation of MRSA+‡ vs do nothing.<br><br>[e] Universal PCR and isolation of MRSA+‡ vs. do nothing.<br><br>[f] Universal pre-emptive isolation‡ vs. do nothing.	Dominant strategy ($/QALY).<br><br>$11,005/QALY.<br><br>$8,114/QALY<br><br>$74,114/QALY.<br><br>$80,159/QALY<br><br>$246,302/QALY.
Robotham[58]	2016	GBR	MRSA	All	Screening strategies using a chromogenic agar test at hospital admission (checklist-activated screening, high-risk specialty-based screening) accompanied by decolonisation and isolation‡ vs. no screening.	Dominated strategy ($/QALY).
Voermans[59]	2019	USA	ABR	Sepsis/LRTI	(PCT)-guided decision algorithm to guide antibiotic prescription‡ vs. standard of care.	Dominant strategy ($/patient diagnosed with ABR bacteria avoided).
You[60]	2012	HKG	MRSA	NICU	Active surveillance (PCR) plus decolonisation‡ vs. active surveillance.	Dominant strategy ($/percentage point reduction in mortality and infection rates).
You[61]	2018	HKG	CRE	ICU	Test-guided selective digestive decontamination‡ vs. no screening.	$688/QALY.
Zboromyrska[62]	2016	ESP	MRSA	BSI	PCR-based assay (GeneXpert) for MRSA detection‡ vs. compared to standard blood culture methods.	$243/LY.

Notes: Costs were calculated in 2022 USDs. ABR=Antibiotic-resistant bacteria. AST=Antimicrobial susceptibility testing. CAN=Canada. CSSSI=complicated skin and skin structure infections. CMS+IMI=Colistin plus imipenem. CNS=Carbapenem-non-susceptible. CPE=Carbapenemase-producing Enterobacteriaceae. CRE=Carbapenem-resistant Enterobacteriaceae. DPO=Dual priming oligonucleotide. FRA=France. DEU=Deutschland or Germany. ICER=Incremental cost-effectiveness ratio. ICU=Intensive Care Unit. IMI/REL=Imipenem/cilastatin/relebactam. IRN= IRAN. KOR= Korea. L= Linezolid. LOS=Length of hospital stay. NLD=The Netherlands. QALYs=Quality-adjusted life years. PCR=Polymerase chain reaction. SD=Standard Deviation. CHE= Switzerland. ESP, Spain. GBR=Great Britain or United Kingdom. KSA= Kingdom of Saudi Arabia. HKG= Hong Kong. TW=Taiwan. USA=United States of America. VRE=Vancomycin-resistant enterococci. IAI=Intrabdominal infections. CR-GN=Carbapenem resistant Gram-negative bacteria. EU= European Union. CRO=Carbapenem-resistant organisms. CLRHP=Clarithromycin-resistant Helicobacter pylori. PRSP= Penicillin-resistant *Streptococcus penumoniae*. MDR=Multidrug resistant bacteria. LRTI= Low respiratory tract infections.

BSI=Bloodstream infections. [⁑]Mupirocin treatment comparing twice a day during two weeks with no follow-up verification (protocol A) versus all patients who received mupirocin (protocol B) for treating post-surgical infections in cardiac surgery. ICER=Incremental cost-effectiveness ratio. [‡]Evaluated strategy (new intervention); ICERs=(cost intervention – cost comparator)/(efficiency intervention – efficiency comparator). A dominant strategy is one in which the incremental cost of the intervention is less than the comparator and the incremental efficacy is greater than the comparator. QALY= Quality adjusted life year. ICU=Intensive care unit. NICU=Neonatal intensive care unit. vs.=versus. HAI= Hospital-acquired infections. LY=Life year.

Allel K, *et al. BMJ Glob Health* 2024; 9:e013205. doi: 10.1136/bmjgh-2023-013205

Table SM7. Characteristics of the included studies (n=59)

First author	Perspective	Type of study	WTP threshold	Discount rate	Time horizon	Source of effectiveness & costs	Year of the EE
**I. Pharmaceutical interventions** (N= 32 studies)							
Bianchini[3]	Health system	CEA, decision tree	$100,000	3%	Lifetime	Literature and RED BOOK[63]	Not stated
Bolaños-Diaz[4]	Health system	CEA, Markov model	$7,200	3%	5 years	Literature and hospital data on costs	Not stated
Bounthavong[5]	Health system	CEA, decision tree model	WTP range, no specific	Not stated	Not stated	Literature and RED BOOK[63]	Not stated
Bounthavong[6]	Health system	CEA, decision tree model	WTP range, no specific	Not applied	15-16 days	Literature and health economic resource centre and decision support services.	2009
Cara[7]	Hospital	CEA, decision tree	Not stated	Not applied	Days in treatment until failure	Hospital outcomes and costs based on a patient-level study	2016
Collins[8]	Payer	CEA, decision tree	$100,000/ QALY	3%	15 years	The ZEPHyR trial and literature.	2014
De Cock[9]	Health system	CEA, decision tree	$52,200	None	11 days	RCT and drug costs insurance reimbursement price and expert panel.	2006
De Cock[10]	Hospital	CEA, decision tree	Not stated	None	Time to cure	RCT and literature.	2006
Goudarzi[11]	Health system	CEA, decision tree	WTP range, no specific	5.8%	5 years	Literature and tariffs from Iran Health System	2022
Gutierrez[12]	Payer	CEA, decision tree	$15,121	3%	30 days and lifetime	Chilean National Reports, Ministry of Health, and Financial entity entrusted to collect, manage and distribute state funds for health	2020
Kong[13]	Health system	CEA, decision tree	$ 12,528/ QALY	5%	5 years	Literature and Yaozh database that collects successful biding prices of drugs	2021
Laohavaleeson [14]	Hospital	CEA, decision tree	$79,750	None	12 days	ATLAS trial outcomes and DRG-specific hospital costs	2006
Lin[15]	Payer	CEA, decision tree	Not stated	Not stated	7-30 days after end of treatment	The ZEPHyR trial and National Health Insurance database (drug costs)	Not stated
McKinnon[16]	Hospital	CUA, mean comparison	Not stated	None	35 days	RCT and nationally representative hospital costs	2006
Mennini[17]	Health system	CEA, decision tree	$21,322/ QALY	3%	5 years	Clinical inputs from phase 3, RCT TANGO II and costs from the Italian official drug pricing list and legislation	Not stated
Mullins[18]	Health system	CEA, decision tree	Not stated	None	11 days	RCT and health insurance claims data	2003
Niederman[19]	Payer	CEA, piggyback and mean comparison	$130,000	None	30 days	ZEPHyR study and literature.	2011
Patel[20]	Payer	CEA, decision tree	$195,804	None	4 weeks	Literature, expert opinion and DRG data	2012
Patel[21]	Payer	CEA, decision tree	$152,400	None	4 weeks	RCT, expert opinion and literature.	2012
Prabhu[22]	Health system	CEA, decision tree and Montecarlo simulation	$39,430	None	Lifetime	RCT and Healthcare cost and utilisation project (HCUP)	2013
Rubio-Terres[23]	Health system	CEA, decision tree	$21,739	7.5%	14-15 days	Literature, Spanish healthcare costs database and General	2011

						Counsel of Official Colleges of Pharmacists.	
Salas[24]	Health system	CEA, decision tree	Not stated	Not applied	14 days	RCT and hospital accounts	Not stated
Schurmann[25]	Hospital and health system	CEA, decision tree	$179,861	None	29 days	RCT, literature and DRG data	2003
Simon[26]	Health system	CEA, decision tree & Markov	$100,000-$150,000/ QALY	3%	5 years	Literature and U.S. Department of Veterans Affairs Federal Supply Schedule.	2017
Tan[27]	Payer	CEA, decision tree	Not stated	None	4 weeks	Trial literature and clinical expert panel	Not stated
Varon[28]	Health system	CEA, decision tree	$3,522	Not applied	30 days	Literature and ISS 2001 rate manual for procedures and SIS-MED (report January-December 2013)	2013
Varon-Vega[29]	Health system	CEA, decision tree	$2,791	None	7-14 days	Colombian manual tariffs and official databases	2019
Vlachaki[30]	Health system	CEA, decision tree	$29,031 and $43,547	3.5%	5 years	British National Formulary, NHS reference costs and literature.	2020
Vu[31]	Health system	CEA, decision tree	$45,789	None	7 days	Federal Supply Schedule, other government agencies (Medicare reimbursements) and literature	2019
Von Dach[32]	Health system	CEA, decision tree	$67,480	Not applied	Duration of therapy until 6 weeks after	RCTs, literature and wholesale prices of generic drugs.	2016
Wan[33]	Payer	CUA, mean differences and bootstrap simulations	Not stated	Not stated	7–30 days after the end of treatment	The ZEPHyR trial, healthcare resource utilisation and literature	2012
Yang[34, 35]	Payer	CEA, decision tree and Markov model	$113,000–169,500	3%	28 days	Literature and red book online database for drug costs.	2020
**II. Non-pharmaceutical interventions** (N= 27 studies)							
Brown[36]	Hospital	CEA, decision tree	$4,669 (EU) & $3,264 (USA)	3%	Not stated	Literature and hospital accounts for microbiological samples	2009
Cho[37]	Hospital	CEA, cost comparison and mean differences	Not stated	Not stated	Not stated	Hospital costs and protocol	Not stated
Dymond[38]	Health system	CEA, decision tree	Not stated	None	12 months	Cambridge University Hospitals NHS Foundation and literature	2010
Gidengil[39]	Hospital	CEA, Markov microsimulation model	$3,015 per colonisation averted and $11,306 per death averted	3%	1 year	Literature and expert consensus	2013
Ho[40]	Health system	CEA, Markov model	$49,149	3%	2 and 10 days	Literature and costs from the largest public health care organization (hospital authority)	2014
Hubben[41]	Hospital	CEA, discrete event simulation model	Not stated	3%	15 years	Literature, bureau of labour statistics and hospital costs	2007
Jayaraman[42]	Hospital	CEA, decision analytic model (tree)	$18,215 and $28,623 per transmission averted.	Not applied	6 months	Literature and estimates excess costs from a MDR outbreak in hospitals	2011

Kang[43]	Hospital	CEA, decision tree	Not stated.	None	Hospital stay long	Framework and literature	2009
Lapointe-Shaw[44]	Hospital	CEA, Markov model	$122,000 per QALY	3%	Not stated	WHO-CHOICE and literature	2016
Lee[45]	Hospital	CEA, Markov model	Not stated	Not applied	Not stated	Literature	2001
Lee[46]	Payer	CEA, decision tree with Montecarlo simulations	$63,733 per QALY	Not stated	Not stated	Literature and Healthcare Cost and Utilization Project National Inpatient Sample.	Not stated
Lee[47]	Societal and payer	CEA, decision analytic stochastic model (tree)	$13,600	3%	Not stated	Human mortality dataset and literature	2008
Lin[48]	Health system	CEA, metapopulation transmission model	Not stated	None	Not stated	Maryland health services cost review commission and literature	Not stated
Luangasanatip [49]	Hospital	CUA, metapopulation transmission model	$5,902/QALY	3%	Lifetime	Literature and hospital data	2016
Mac[50]	Hospital	CEA, microsimulation model	Not stated	1.5%	1 year at hospital and lifetime	Literature	2017
Murthy[51]	Hospital	CEA, decision analysis (tree)	Not stated	Not stated	Hospitalis ation period	Hospital's cost accounting system and literature	2006
Nelson[52]	Health system	CEA, decision tree	Not stated	Not stated	Inpatient's stay	Literature	Not stated
Nelson[53]	Health system	CEA, decision tree and budget impact model	Not stated	3%	29 years	Literature	2013
Nelson[54]	Health system	CEA, simulation model	WTP range, no specific	3%	8 years	Literature and Nationwide Inpatient Sample database	2019
Penno[55]	Hospital	CEA, decision tree	$6,500 per life saved	Not stated	Not stated	WHO and clinical laboratory data	2011
Puzniak[56]	Hospital	CBA, cost and outcome comparison	Not stated	Not stated	Not stated	Literature and line-item reports from the hospital's microbiology database	Not stated
Robotham[57]	Health system	CEA, mathematical individual-based model of transmission	WTP range, no specific	Not stated	Not stated	Literature, National Health Service data and primary data	Not stated
Robotham[58]	Health system	CEA, mathematical model of transmission	$62,500 per QALY	Not stated	Five years	National health system (NHS) and literature	2011
Voermans[59]	Societal and hospital	CEA, decision tree	Not stated	Not applied	Length of hospital stay (<1 year)	Hospital data and literature	2019
You[60]	Health system	CEA, decision tree	Not stated	Not stated	Not stated	Literature and microbiology laboratory of a public hospital in Hong Kong	Not stated
You[61]	Health system	CEA, Markov model	$50,123	3%	Not stated	Literature and local hospital costs (health authority)	2015
Zboromyrska[62]	Hospital	CEA, decision tree	WTP range, no specific	3%	Length of hospital stay	Literature and hospital data on prevalence	Not stated

Notes: WTP= Willingness to pay. EU= European union. USA= United States of America. QALY= Quality-adjusted life year. EE= Economic evaluation. DRG= Diagnostic-related group. RCT= randomised controlled trial. CEA= Cost-effectiveness analysis. CUA= Cost-utility analysis. Costs are reported in 2022 USD$. CBA= Cost-benefit analysis.

Table SM8. Unit costs per study for pharmacological interventions (in 2022 USDs)

I. Pharmaceutical interventions (A): Patients with MRSA or suspected MRSA investigating Cellulitis or Complicated Skin and Skin Structure Infections (cSSSI) treated with linezolid versus vancomycin

	**Linezolid costs ($)**						**Vancomycin costs ($)**					
**Article**	**ICU ward**	**General ward**	**Tests**	**Drugs**	**Additional**	**Total**	**ICU ward**	**General ward**	**Tests**	**Drugs**	**Additional**	**Total**
**Bounthavong, 2009[5]**	NS	$1565 Ward per day	NS	$256 (iv) per day<br>$200 (oral) per day	$53 microbiology culture per day<br>$11 platelet monitoring per day	$13938	NS	$1565 Ward per day	NS	$11 (iv) per day	$8 vancomycin labs per day<br>$53 microbiology culture per day<br>$11 platelet monitoring per day	$34076
**Bounthavong, 2011[6]**	NS	$2687 per day	NS	$303 (iv) per day<br>$232 (oral) per day	$55 Microbiological culture, per day<br><br>$12 Platelet monitoring, per day	$22752	NS	$2687 per day	NS	$18 (iv) per day	$55 Microbiological culture, per day<br><br>$12 Platelet monitoring, per day	$29825
**De Cock, 2009a[10]**	$1095 ICU without ventilator, per day<br><br>$1594ICU weighted average, per day	$322 Ward per day	$655	$332 (iv), per day.<br><br>$322 (oral), per day.	$505 Isolation, per day.<br><br>$27 Infusion (iv) longer than 30 minutes<br><br>$371 Adverse events	$23,357	$1095 ICU without ventilator, per day<br><br>$1594ICU weighted average, per day	$322 Ward per day	$803	$89 (iv) per day	$505 Isolation, per day.<br><br>$27 Infusion (iv) longer than 30 minutes<br><br>$371 Adverse events	$20722
**McKinnon, 2006[16]**	$1512 per day	$617 per day	NS	$182 (iv), per day<br><br>$134 (oral), per day	$68 Intravenous administration/dose<br><br>$803 Step-down; per day	$6492	$1512 per day	$617 per day	NS	$35 (iv), per day	$68 Intravenous administration/dose<br><br>$803 Step-down; per day	$7988
**Schurmann, 2009[25]**	NS	$336 per day	NS	$304 (iv), per day<br>$295 (oral), per day	$530 Isolation, per day<br><br>$26 Intravenous infusion, per day	$11013	NS	$356 per day	NS	$130 (iv), per day	$530 Isolation, per day<br><br>$26 Intravenous infusion, per day	$13188

					$68 GP, per home visit.<br><br>$65 Specialist, per consultation<br><br>$63 GP, per office visit<br><br>$489 Other inpatient (test and adverse events)<br><br>$2490 Post discharge (outpatient antibiotic drugs, test, visit)						$68 GP, per home visit.<br><br>$65 Specialist, per consultation<br><br>$63 GP, per office visit<br><br>$738 Other inpatient (test and Adverse events)<br><br>$1911 Post discharge (outpatient antibiotic drugs, test, visit)	
**I. Pharmaceutical interventions (B):** Patients with MRSA or suspected MRSA investigating Cellulitis or Complicated Skin and Skin Structure Infections (cSSSI) Linezolid treated with daptomycin												
Linezolid. Cost ($)							Daptomycin cost ($)					
**Article**	**ICU ward**	**General ward**	**Tests**	**Drugs**	**Additional**	**Total**	**ICU ward**	**General ward**	**Tests**	**Drugs**	**Additional**	**Total**
**Bounthavong, 2011[6]**	NS	$2687 per day	**NS**	$303 (iv) per day<br>$232 (oral) per day	$55 Microbiological culture, per day<br><br>$12 Platelet monitoring, per day	$ 22752	NS	$2687 per day	NS	$344 (iv) per day	$55 Microbiological culture, per day<br><br>$12 Platelet monitoring, per day	$26079
**I. Pharmaceutical interventions (C):** Patients with MRSA or suspected MRSA investigating Cellulitis or Complicated Skin and Skin Structure Infections (cSSSI) Telavancin versus Vancomycin												
Telavancin cost ($)							Vancomycin cost ($)					
**Article**	**ICU ward**	**General ward**	**Tests**	**Drugs**	**Additional**	**Total**	**ICU ward**	**General ward**	**Tests**	**Drugs**	**Additional**	**Total**
**Laohavaleeson, 2008[14]** (Telavancin versus Vancomycin)	NS	NS	NS	$18	$144 Study drug<br><br>$528 Additional antibiotic	$11801	NS	NS	NS	$18	$144 Study drug<br><br>$568 Additional antibiotic<br><br>$68 Drug monitoring	$10345

18

I. Pharmaceutical interventions (D): Patients with MRSA or suspected MRSA investigating Nosocomial Pneumonia treated with linezolid versus vancomycin

Article	Linezolid costs ($)						Vancomycin costs ($)					
**Collins, 2015[8]**	NS	NS	NS	$283 (iv) per day<br><br>$235 (oral) per day	$16544 Attributable cost, Nephrotoxicity<br><br>$24047 Attributable cost, Thrombocytopenia<br><br>$2047 Attributable cost, Pneumonia	$27009	NS	NS	$10	$31	$16544 Attributable cost, Nephrotoxicity<br><br>$24047 Attributable cost, Thrombocytopenia<br><br>$2047 Attributable cost, Pneumonia	$2598
**De Cock, 2009b[9]**	$2401 per day	$391 per day	$171 Biochemistry monitoring test, per unit	$104 (iv or oral)	$255 Monitoring test (biochemical, hemogram, C- reactive protein, other drugs<br><br>$104 Co-medications<br><br>$120Treatment Acute Encephalitis Syndrome (AEs)<br><br>$341 Post-discharge (visit and test)	$12989	$2401 per day	$391 per day	$171 Biochemistry monitoring test, per unit<br><br>$1079	$7 (iv)	$255 Monitoring test (biochemical, hemogram, C-reactive protein, other drugs<br><br>$159 Co-medications<br><br>$149 Treatment Acute Encephalitis Syndrome (AEs)<br><br>$509 Post-discharge (visit and test)	$14657
**Lin, 2016[15]**	$474 per day	$87 per day	NS	$1252	$2 Lab work (serum creatinine levels)<br>$	$6900	$474 per day	$87 per day	NS	$263	$2 Lab work (serum creatinine levels)<br><br>$12 Lab work (serum vancomycin levels)	$6474
**Mullins, 2006**	NS	NS	NS	$2949, per day	NS	$33331	NS	NS	NS	$3132 per day	NS	$33511 per day
**Niederman, 2014[19]**	$3520 per day	$1645 per day	$44	$131 (iv)	$2133 Mechanical ventilation<br><br>$2449 Study drugs	$54905	$3520 per day	$1645 per day	Laboratory test: $47	$7 (iv)	$2086 Mechanical ventilation<br><br>$306 Study drugs<br><br>$604 Dialysis	$54774

					$132 Dialysis							
**Patel, 2014a [21]**	$4078 to adjusted to received therapy.<br>*$4065 per day	$2917 total adjusted to received therapy.<br>*$2349 per day	$78	$25	$205 administration<br>$2344 Physician/attending visit.<br>$1353 Lab work<br>$2573 Serious adverse event<br>$2224 Mechanical ventilation	$54940	$41326 total adjusted to received therapy.<br>*$4065 per day	$4194 total adjusted to received therapy.<br>*$2349 per day	$43	$888	$ 217 administration<br>$2488 Physician/attending visit.<br>$1482 Lab work<br>$3155 Serious adverse event<br>$2171 Mechanical ventilation	$55920
**Patel, 2014b[20]**	*$1878 ICU +mechanical ventilation, per day	*$1077 Ward + isolation, per day:	NS	$87 (iv)	NS	$23025 Total base case inpatient	*$1878 ICU +mechanica l ventilation, per day	*$1077 Ward + isolation, per day:	NS	$14 (iv)	NS	$23212 Total base case inpatient
**Tan, 2014[27]**	$2093 Beijing<br>$2415 Guangzhou<br>$22157 Nanjing<br>$1530 Xi`an	$277 Beijing<br>$293 Guangzho u<br>$283 Nanjing<br>$223 Xi`an	NS	$143 per vial	NS	$24716 Beijing<br>$28012 Guangzhou<br>$25376 Nanjing<br>$18945 Xi`an	$2093 Beijing<br>$2415 Guangzhou<br>$22157 Nanjing<br>$1530 Xi`an	$277 Beijing<br>$293 Guangzhou<br>$283 Nanjing<br>$223 Xi`an	NS	$46, per vial	NS	$24700 Beijing<br>$28025 Guangzhou<br>$25375 Nanjing<br>$18802 Xi`an
**Varon, 2014[28]**	$856 Stay (ICU and standard room)		NS	$1097	$4452 Management of kidney failure	1521	$856 Stay (ICU and standard room)		NS	$83	NS	1166

					$83 Management of thrombocytopenia							
**Wan, 2016[33]**	$1719 Beijing<br>$1176 Xi`an	$287 Beijing<br>$231 Xi`an		$151 (iv) Beijing<br>$151 (iv) Xi`an	$200 Mechanical ventilator Beijing, per day<br>$160 Mechanical ventilator Xi`an, per day<br>$1066 Continuous renal replacement therapy Beijing, per day<br>$1583 Continuous renal replacement therapy Xi`an, per day	$ 26506 Beijing<br>$30320 Guangzhou<br>$27450 Nanjing<br>$19796 Xi`an	$1719 Beijing<br>$1176 Xi`an	$287 Beijing<br>$231 Xi`an	$32 Beijing and Xi`an	$96 (iv) Beijing<br>$96 (iv) Xi`an	$4452 Management of kidney failure<br>$83 Management of thrombocytopenia<br>$200 Mechanical ventilator Beijing, per day<br>$160 Mechanical ventilator Xi`an, per day<br>$1066 Continuous renal replacement therapy Beijing, per day<br>$1583 Continuous renal replacement therapy Xi`an, per day	$25852 Beijing<br>$29804 Guangzhou<br>$26922 Nanjing<br>$19260 Xi`an
**I. Pharmaceutical interventions (E):** Patients with MRSA or suspected MRSA investigating Nosocomial Pneumonia treated with low dose of colistin and high dose of colistin												
Low dose of colistin Cost ($)							High dose of colistin treatment Cost ($)					
**Article**	**ICU ward**	**General ward**	**Tests**	**Drugs**	**Additional**	**Total**	**ICU ward**	**General ward**	**Tests**	**Drugs**	**Additional**	**Total**
**Cara, 2018[7]**	$3850 per day	$3908 per day	$836 CBC per day	$932 per day	$1045 GW nurse visits, per day<br>$770 ICU nurse visits, per day<br>$281 Laboratory cultures, per day	$22912	$5587 per day	$3400 per day	$842 CBC, per day	$624, per day	$1052 GW nurse visits, per day<br>$1117 ICU nurse visits, per day<br>$188 Laboratory cultures, per day	$20390
**I. Pharmaceutical interventions (F):** Patients with MRSA or suspected MRSA investigating Nosocomial Pneumonia treated with Ceftazidime-avibactam vs. colistin-based treatment.												
Ceftazidime-avibactam cost ($)							Colistin-based cost ($)					

Article	ICU ward	General ward	Tests	Drugs	Additional	Total	ICU ward	General ward	Tests	Drugs	Additional	Total
**Bolaños-Diaz, 2022[4]**	NS	NS	NS	$12240 per day	$267 Hospitalization costs, per day<br><br>$19999 Long-term care, per year<br><br>$19094 Nephrotoxicity, Chronic dialysis, per year<br><br>$2203 Nephrotoxicity, With RRT<br><br>$12240 Nephrotoxicity, Without RRT	$28764				$163 per day	$267 Hospitalization costs, per day<br><br>$19999 Long-term care, per year<br><br>$19094 Nephrotoxicity, Chronic dialysis, per year<br><br>$2203 Nephrotoxicity, With RRT<br><br>$12240 Nephrotoxicity, Without RRT	$16322
**Simon, 2019[26]**	NS	NS	NS	$1028 per day	$100355 chronic dialysis, per year<br><br>$105113 long-term care, per year<br><br>$26722 long-term health care for sepsis, first year<br><br>$8971 long-term health care costs of sepsis, subsequent year	$173493	NS	NS	NS	$29 per day	$100355 chronic dialysis, per year<br><br>$105113 long-term care, per year<br><br>$26722 long-term health care for sepsis, first year<br><br>$8971 long-term health care costs of sepsis, subsequent year	$120768
**Varon-Vega, 2022[29]**	$332	$37	NS	$43	$452 Adverse event, Renal failure<br><br>$1269 Adverse event, Dialysis	8781	$332	$13	NS	$13	$452 Adverse event, Renal failure<br><br>$1269 Adverse event, Dialysis	$5264
**I. Pharmaceutical interventions (G):** Patients treated with other intervention types for MRSA and gram-negative infections including carbapenem non-susceptible infections												

Intervention 1 cost ($)							Intervention 2 cost ($)					
**Article**	**ICU ward**	**General ward**	**Tests**	**Drugs**	**Additional**	**Total**	**ICU ward**	**General ward**	**Tests**	**Drugs**	**Additional**	**Total**
**Bianchini, 2022[3]** (New beta-lactam beta-lactamase Inhibitor antibiotics‡ vs. colistin treatment)	NS	NS	NS	$1259 Meropenem-vaborbactam, per day<br><br>$870 Ceftolozane-tazobactam, per day<br><br>$1361 Imipenem-relebactam, per day<br><br>$40 Polymyxin, per day<br><br>$207 Meropenem, per day	NS	$17172	NS	NS	NS	$8	NS	$3710
**Goudarzi, 2023[11]** (Ceftazidime avibactam vs. colistin treatment.)	NS	NS	NS	$649	$598 Long term care<br>$901 nephrotoxicity without renal replacement therapy<br><br>$9764 Nephrotoxicity with renal replacement therapy	$885	NS	NS	NS	$445	$598 Long term care<br>$901 nephrotoxicity without renal replacement therapy<br><br>$9764 Nephrotoxicity with renal replacement therapy	$460
**Gutierrez, 2021[12]** **(**Ceftazidime/ avibactam vs.	$240	$58	NS	$128, per vial	$240 Prevention of kidney failure<br><br>$1256 Dialysis, per month	$9566	$240	$58	NS	$8 Meropenem<br><br>$15 Colistin	$240 Prevention of kidney failure<br><br>$1256 Dialysis, per month	$6423

colistin+ meropenem treatment)					$24 Creatinine<br>$2 Ureic nitrogen<br>$4 Hemogram<br>$0.2 Plasma electrolytes						$24 Creatinine<br>$2 Ureic nitrogen<br>$4 Hemogram<br>$0.2 Plasma electrolytes	
**Kong, 2023[13] (**Ceftazidime-avibactam vs. polymyxin B (PMB) monotherapy)	NS	NS	NS	$606 per day	$2483 Long-term care<br>$5715 Nephrotoxicity without renal replacement therapy in hospital<br>$11955 Nephrotoxicity with renal replacement therapy in hospital<br>$30746 Hemodialysis, per year	$23726934	NS	NS	NS	$667 per day	$2483 Long-term care<br>$5715 Nephrotoxicity without renal replacement therapy in hospital<br>$11955 Nephrotoxicity with renal replacement therapy in hospital<br>$30746 Haemodialysis, per year	$23514366
**Mennini, 2021[17]** Vaborem (meropenem-vaborbactam) versus best available treatment	NS	NS	NS	$1141 Carbapenems	$8206 Hospital acquired pneumonia (HAP)/ Ventilation associated pneumonia (VAP)<br>$3856 Complicated urinary tract infections (cUTI)	$3287	NS	NS	NS	$3428 Ceftazidime-Avibactam<br>$642 Colistin<br>$23 Aminoglycosides	$8206 Hospital acquired pneumonia (HAP)/ Ventilation associated pneumonia (VAP)<br>$3856 Complicated urinary tract infections (cUTI)<br>$4975 Complicated intra-abdominal infections (cIAI)	$ 2121

					$4975 Complicated intra-abdominal infections (cIAI)<br><br>$7844 Bloodstream infections (BSI)						$7844 Bloodstream infections (BSI)	
**Rubio-Terres, 2012[23]** (Daptomycin vs. vancomycin treatment.)	NS	NS	NS	$160	$2 Sodium chloride 0.9% (1 bag of 50 mL)<br><br>$1 Sterile water for injection (1 ampoule of 20 mL)<br><br>$1324 Admission to the Infectious Diseases Service (1 day)<br><br>$802 Admission to the Internal Medicine Service (1 day)<br><br>$0.5 IV administrations by a nurse (1 minute of work day)	$ 21359 per patient	NS	NS	NS	$12	$2 Sodium chloride 0.9% (1 bag of 50 mL)<br><br>$1 Sterile water for injection (1 ampoule of 20 mL)<br><br>$1324 Admission to the Infectious Diseases Service (1 day)<br><br>$802 Admission to the Internal Medicine Service (1 day)<br><br>$0.5 IV administrations by a nurse (1 minute of work day)	$ 21995 per patient
**Salas, 2016[24]** (Protocol A versus B) [24]	*$4258 per day	*$2063 per day	NS	$1195 Screening and treatment<br><br>*$10 Mupirocin ointment	*$113 Nurse, per hour<br><br>*$74 Nursing assistant, per hour<br><br>*$0.04 Chlorhexidine (sponge)	$49683 per patient	*$4258 per day	*$2063 per day	NS	$2894 Screening and treatment:<br><br>*$10 Mupirocin ointment	**$113 Nurse, per hour<br><br>*$74 Nursing assistant, per hour<br><br>*$0.04 Chlorhexidine (sponge)<br><br>*$0.35 Syringe 2ml 2 bodies	$47254 per patient

					$0.35 Syringe 2ml 2 bodies<br><br>$0.04 Non-sterile latex glove<br><br>*$8 Dish culture						*$0.04 Non-sterile latex glove<br><br>*$8 Dish culture	
**Prabhu, 2017[22]** (CTM versus PT) [22]	*$659 per day	*$549 per day	NS	$1161 Total cost per patient	NS	$4340 per patient considering hospitalisation and drugs costs	*$659 per day	*$549 per day	NS	$275 Total cost per patient	NS	$3656 per patient considering hospitalisation and drugs costs
**Vlachaki, 2021[30]** **(**Vaborem (meropenem-vaborbactam) vs. best available treatment)	$2748 per unit	$525 per unit	NS	$ 4222	$96 Adverse events<br><br>$2793 Clinical failure<br><br>$143 Nephrotoxicity<br><br>$61 renal replacement therapy (in hospital)<br><br>$49 renal replacement therapy<br><br>$170 Chronic renal replacement therapy	$66338 Total cost for long-term	$2748 per unit	$525 per unit	NS	$1202	$816 Adverse events<br><br>$5043 Clinical failure<br><br>$1253 Nephrotoxicity<br><br>$492 renal replacement therapy (in hospital)<br><br>$330 renal replacement therapy<br><br>$1149 Chronic renal replacement therapy	$58656 Total cost for long-term
**Yang, 2022[34]**	*$4472 per day	*$2618 per day	NS	$12833 Total cost:	$4550 Adverse events	102622	*$4472 per day	*$2618 per day	NS	$2620 Total cost	$16145 Adverse events<br><br>$95097 Hospital recourse	$114156

(IMI/REL versus CSM+IMI) [34]					$84813 Hospital recourse: $92,153<br><br>$426 Long-term monitoring						$294 Long-term monitoring	

**I. Pharmaceutical interventions (H):** Patients with MRSA tackling interventions with BSI Daptomycin vs. linezolid 4-weeks treatment												
**Article**	**ICU ward**	**General ward**	**Tests**	**Drugs**	**Additional**	**Total**	**ICU ward**	**General ward**	**Tests**	**Drugs**	**Additional**	**Total**
	**Intervention 1 Costs ($)**						Intervention 2 Costs ($)					
**Vu, 2021[31]** Daptomycin vs. linezolid 4-weeks treatment.	NS	$3576	NS	$89 Daptomycin per day	$0.4 Monitoring per Daptomycin: 1Creatinine phosphokinase test per week, per day	$33918	NS	$3576	NS	$35 (iv) per day $3 (oral) per day	NS	$33004
**Vu, 2021[31]** (Linezolid vs. vancomycin 4-weeks treatment)	NS	$3576	NS	$35 (iv) per day $3 (oral) per day	NS	$33004	NS	$3576	NS	$3 per day	$2 Monitoring per Vancomycin: 1 trough every 3 day, per day	$34414
**Vu, 2021[31]** (Linezolid vs. ceftaroline/daptomycin 4-weeks treatment)	NS	$3576	NS	$35 (iv) per day $3 (oral) per day	NS	$33004	NS	$3576	NS	$367 Ceftaroline per day<br><br>$89 Daptomycin per day	$0.4 Monitoring per Daptomycin: 1Creatinine photsphokinese test per week, per day	$33918

**I. Pharmaceutical interventions (I):** Patients with MRSA tackling interventions Trimethoprim-sulfamethoxazole + rifampicin vs. linezolid												
**Trimethoprim-sulfamethoxazole+ rifampicin cost ($)**							**Linezolid cost ($)**					
**Article**	**ICU ward**	**General ward**	**Tests**	**Drugs**	**Additional**	**Total**	**ICU ward**	**General ward**	**Tests**	**Drugs**	**Additional**	**Total**
**Von Dach, 2017[32]**	NS	NS	NS	$6 (iv) trimethoprim-sulfamethoxazole	$23 adverse drug reaction<br><br>$2 IV material	$165	NS	NS	NS	$104 (iv) $106 (oral)	$11 adverse drug reaction<br><br>$2 IV material	$2865

			$1 (iv) trimethoprim-sulfamethoxazole<br><br>$42 (iv) rifampicin<br><br>$4 (oral) rifampicin			

Notes: ICU, intensive care unit; tests: included diagnostic tests during inpatient stay; drugs: included drug acquisition cost only; additional: additional costs including monitoring costs, drug administration costs, isolation costs; NS, not stated, i.e., the study did not explicitly state this data. AEs, Acute encephalitis syndrome. CI, Confidence intervals. SD, Standard deviation. LOS, Length of hospital stay. Iv, Intravenous. GP, General practitioner. Where standard deviation or confidence intervals were reported, these have been included. Pd, der diem. Costs were calculated in 2022 USDs. All costs were inflated using the following website (http://eppi.ioe.ac.uk/costconversion/default.aspx). Drug acquisition costs were either found from nationally representative wholesale values or from hospital purchasing departments. Additional costs, whenever reported, ranged from isolation costs for intensive care unit (ICU) wards, monitoring and drug administration and diagnostic costs, as part of moving from empirical therapy to targeted antibiotics. *Generic values used for wards or ICU beds, ventilator, and tests, regardless therapy, or treatment.

Allel K, *et al. BMJ Glob Health* 2024; 9:e013205. doi: 10.1136/bmjgh-2023-013205

Table SM9. Unit costs per study for non-pharmaceutical interventions (in 2022 USDs)

Article	Unit Costs				Total costs
	Staff	Hospital	Test/intervention	Additional costs	
**Brown, 2010[36]**	Performing the test and specimen collection $32	NS	PCR test USA $79<br><br>PCR test UE $89<br><br>Total cost PCR. USA $29859<br>Total cost PCR. UE $22999	The weighted mean treatment. US $41199<br><br>The weighted mean treatment. EU $60366	NS
**Cho, 2019[37]**	Physicians visit $16	Endoscopy without sedation: $67<br><br>Endoscopy with sedation: $165	*Helicobacter pylori* diagnosis screening for rapid urease test (RUT): $10<br><br>*Helicobacter pylori* diagnosis screening for DPO-PCR testing: $69<br><br>*Helicobacter pylori* diagnosis screening biopsy: $9<br><br>*Helicobacter pylori* diagnosis screening endoscopy forceps: $21	Urea breath test: $32	Clarithromycin-based triple therapy first -line treatment, per patient: $59820<br><br>Clarithromycin-based triple therapy second-line treatment, per patient: $62412<br><br>Tailored therapy using DPO-PCR, first-line therapy, per patient: $37468<br><br>Tailored therapy using DPO-PCR, second-line therapy, per patient: $37791
**Dymond, 2020[38]**	NS	NS	Genome sequenced, per unit: $108<br>Total genome sequences WGS+CP: $77183<br><br>Screening positive, per unit: $9<br>Screening negative, per unit: $5	Symptomatic MRSA, per case: $18617<br><br>Asymptomatic MRSA, per case, per case: $418<br><br>MRSA-related treatment WGS, annual hospitalized cohort: $2132431<br><br>Admission screening cost WGS+CP, annual hospitalized cohort: $296419.<br><br>Outbreak investigation screening WGS+CP, annual hospitalized cohort: $42237	Total cost WSP+CP, annual hospitalized cohort: $2545423

**Article**	**Unit Costs**				**Total costs**
	Staff	Hospital	Test/intervention	Additional costs	
				Clinical sampling WGS+CP, annual hospitalized cohort: $554	
**Gidengil, 2015[39]**	NS	NS	Active surveillance cultures test: $15<br><br>Contact precautions per day: $146<br><br>Chlorhexidine gluconate bath per day: $13<br><br>Decolonization (chlorhexidine gluconate + mupirocin) per day: $27	NS	Active surveillance cultures testing plus selective decolonization, per 10000 patients (millions): $6<br><br>Active surveillance cultures testing alone, per 10000 patients (millions): $8<br><br>Universal contact precautions alone, per 10000 patients (millions): $10
**Ho, 2016[40]**	ICU care, per day $3362	NS	PCR : $29	Adequate therapy for CRE infection: $228<br><br>Inadequate therapy for CRE infection: $56	Active surveillance CRE-associated, cost per patient: $1436
**Hubben, 2011[41]**	Take swab by nurse (5 min): $4<br><br>Clinical risk assessment by nurse (5 min): $4<br><br>PCR test cost lab. Technician time, per sample: $1<br><br>Chromogenic clinical lab. technician time, per sample: $7<br><br>Contact precaution additional physician time (10 min), per day: $18		PCR- test cost, per sample: $31<br><br>Chromogenic screening, per sample: $5	Contact precautions material, per day: $16<br><br>Clearing of room (30 min): $62	The investment costs of 'Selective Chromogenic' in a high prevalence setting (m): 11<br><br>The investment costs of 'Selective Chromogenic' in a medium prevalence setting (m): $8<br><br>The investment costs of 'Universal PCR' in a high prevalence setting (m): 21<br><br>The investment costs of 'Universal PCR' in a medium prevalence setting (m): $19

**Article**	**Unit Costs**				**Total costs**
	Staff	Hospital	Test/intervention	Additional costs	
**Jayaraman, 2016[42]**	Total cost nursing, General surgery ICU, per 6 weeks: $ 116813<br><br>Staffing Surge pods, per 6 weeks: $2126<br><br>Total cost nursing, General surgery ICU, per 1 week:$19469<br><br>Staffing Surge pods, per 1 week: $2126	NS	NS	Overall excess costs, per 6 weeks: $41790<br>Overall excess costs, per 6 weeks $195250<br><br>Total Supply renewal, per 6 weeks: $20042<br>Total Supply renewal, per 1 week: $3218	Model program per year: $83581
**Kang, 2012[43]**	Registered Nurse, per hour: $40<br><br>Physician, per hour: $105	NS	Rapid PCR test: $63	Contact precaution: gown, per unit: $1<br><br>Contact precaution: pair of gloves: $0.1	Universal screening strategy: $10248049<br><br>Target screening strategy: $8138164<br><br>None screening strategy: $8494454
**Lapointe-Shaw, 2017[44]**	NS	NS	Screening (PCR): $37<br><br>Screening (swab and conventional culture plating): $13	Isolation, per day: $40<br><br>Attributable cost of pneumonia: $23912<br><br>Attributable cost of bloodstream infection: $18400<br><br>Attributable cost of urinary tract infection: $3432	NS
**Lee, 2005[45]**	Physician ‘wages, per hour: $270<br><br>Healthcare workers’ wages, per hour: $38	Hospitalisation, per day: $1610	Screening, per patient admitted-with current screening practice: $3.<br><br>Screening, per patient admitted-with current screening plus those with a history of renal disease: $3	Isolation cart: $273<br><br>Laboratory, per test: $8<br><br>Extra laboratory per positive results: $ 11	Total cost per patient admitted with current screening practice: $6816<br><br>Total cost per patient with current screening plus those with a history of renal disease: $7770

Article	Unit Costs				Total costs
	Staff	Hospital	Test/intervention	Additional costs	
**Lee, 2009[46]**	NS	NS	Screening per patient admitted with current screening plus those with a hospitalisation in the previous 2-years: $4.<br>Surveillance: $12<br>Decolonization: $131	Wound infection (Hospitalization): $5901<br><br>Graft infection (Hospitalization): $16327<br><br>Amputation (hospitalization): $15022<br><br>Infected stump (hospitalization): $9814<br><br>Line infection (hospitalization): $30972<br><br>Urinary tract infection (hospitalization): $636<br><br>Pneumonia (hospitalization): $16439	Total cost per patient admitted with current screening plus those with a hospitalisation in the previous 2-years l: $6096<br><br>NS
**Lee, 2010[47]**	NS	Hospitalisation, per person (range); $5335-$30717	Universal MRSA Surveillance testing (culture): $13	Vancomycin; $11<br><br>Extra procedures: blood cultures, cardiac surgery, placing patient in contact isolation.(range): $40-$8,835.	Total cost: $7352
**Lin, 2021[48]**	Staffing cost for implementing contact precautions, per patient/day: $59	The average cost for implementation electronic registry per CRE infection: $32,923	Total cost per active surveillance screening test (cultured-based screening): $9<br>Total cost screening (cultured-based screening): $12240	Implementation of the electronic registry, per hospital: $10200<br>IPC bundle per CRE patient: $652	The net cost of interventions: $222360
**Luangasanatip, 2018[49]**	NS	Paediatric ICU, per ward, per year: $728<br><br>Base case, Adult ICU, per ward, per year: $719	NS	Total cost hand hygiene (paediatric ICU), per year: $763<br><br>Total cost hand hygiene (adult ICU), per year: $814	Baseline (hand hygiene compliance 10%) in paediatric ICU: $34302013

Article	Unit Costs				Total costs
	Staff	Hospital	Test/intervention	Additional costs	
					Hand hygiene compliance 20%, in pediatric ICU: $34305035<br><br>Hand hygiene compliance 40%, in pediatric ICU: $34306617<br><br>Hand hygiene compliance 60%, in pediatric ICU: $34307083<br><br>Baseline (hand hygiene compliance 10%) in Adult ICU: $24366979<br><br>Hand hygiene compliance 20%, in Adult ICU: $24371521<br><br>Hand hygiene compliance 40%, in Adult ICU: $24373669<br><br>Hand hygiene compliance 60%, in Adult ICU: $24374285
**Mac, 2019[50]**	Nurse time, per test: $6	Private room, daily: $264	Rectal swab screen: $3<br><br>Culture, positive test: $19<br><br>Culture, negative test: $8	Personal protective equipment, per room visit: $2<br><br>Antibiotics, bacteraemia, per day: $477<br><br>Antibiotics, other infections, per day: $33	NS
**Murthy, 2010[51]**	NS	Cost per surgical bed-day during the study period: $265	Decolonization treatment, mupirocin 2%: $3<br><br>PCR screening: $7<br><br>Standard chromogenic agar culture	Cost of standard chromogenic agar culture : $7	No MRSA screening: $1653<br><br>Universal rapid PCR screening: $1676
**Nelson, 2010[52]**	Total cost of extra nurse and physician time attributable to isolation: $105	NS	Screening: $62<br><br>Decolonization: $37	Isolation: $594<br><br>Chlorhexidine showers: $6	NS

Article	Unit Costs				Total costs
	Staff	Hospital	Test/intervention	Additional costs	
	Physician visit: $93			MRSA infection: $24800<br><br>pair of gloves: $9<br><br>gown: $1	
**Nelson, 2016[53]**	NS	NS	NS	NS	Straight line assumption, Total (Overall costs): $88053741<br><br>Downward trend assumption Total (Overall costs): $59310260
**Nelson, 2021[54]**	Workload for nurses, per day: $71<br><br>Workload for physicians, per day: $9<br><br>workload for other hospital staff, per day: $18<br><br>MRSA Prevention Coordinator, per year: $ 28727<br><br>Laboratory technician, per year: $ 75179	NS	Screening test, per patient: $29<br><br>The total cost of screening on admission (millions): $146	Isolation materials including gowns, gloves, surgical masks, goggles, and isolation laundry double bags, per day: $47<br><br>Cleaning materials, per day: $6<br><br>educational materials first year: $ 6448<br><br>educational materials each subsequent year: $ 1247	NS
**Penno, 2015[55]**	Laboratory technician performing a human immunodeficiency virus, per hour: $7<br><br>Clinical assessment set (10 min), per case: $1	NS	Total Negative blood culture (reagent and supplies, indirect cost, equipment), per test: $14<br><br>Total Positive blood culture (reagent and supplies, indirect cost, equipment), per test: $88	Additional cost, per patient: $31	Total cost generic antimicrobials, per case: $16<br><br>Total cost evidence-based antimicrobials, per case: $32
**Puzniak, 2004[56]**	Nursing time to don and doff gowns, per day: $63	NS	Vancomycin-resistant enterococci-negative test, per unit: $17	Gown, per day: $106<br><br>Gloves, per day: $10<br><br>Hand hygiene, per day: $14	Total cost of policies. Gown period, for patient in ICU: $380312

**Article**	Unit Costs				**Total costs**
	Staff	Hospital	Test/intervention	Additional costs	
			Vancomycin-resistant enterococci positive test, per unit: $34		Total cost of policies. Annualized Gown period, for patient in ICU: $ 253541<br><br>Total cost of policies. Non gown period, for patient in ICU: $149208
**Robotham, 2011[57]**	NS	NS	Screening positive result: $16<br><br>Screening negative result: $10	Swabbing cost: $8<br><br>Contact precaution, general hospital: $30<br><br>Decolonisation, general hospital: $103	No screening, per admission: $23326
**Robotham, 2016[58]**	NS	Hospitalisation Bed (ICU and general ward), per day: $581	Screening positive result: $13<br><br>Screening negative result: $7	Swabbing cost: $6<br><br>Contact precaution, general hospital: $41.<br><br>Treatment, 14 days: $925	No screening, per admission: $4552<br><br>High risk specialties, per admission: $4618<br><br>Checklist activated, per admission: $4686<br><br>High risk specialities +checklist activated, per admission: $4719<br><br>All admissions: $4807<br><br>All admissions + pre-emptive isolation: $4812
**Voermans, 2019[59]**	NS	Hospitalisation general ward, per day: $1383<br><br>Hospitalisation ICU, per day: $2061	PCT test: $102<br><br>CDI test: $99	Isolation, per day: $54<br><br>Mechanical ventilation, per day: $1143<br><br>Antibiotic, per day: $184<br><br>Blood culture: $47	NS

**Article**	**Unit Costs**				**Total costs**
	Staff	Hospital	Test/intervention	Additional costs	
**You, 2012[60]**	NS	Neonatal intensive care unit care, per day: $38	Polymerase chain reaction: $30	NS	Active surveillance plus decolonization in Neonatal Intensive Care Unit: $56280<br><br>Active surveillance alone in Neonatal Intensive Care Unit: $57157
**You, 2018[61]**	NS	ICU-acquired infection: $57<br><br>ICU care, per day: $3244	Polymerase chain reaction test: $30	Oral gentamicin and colistin, per day: $109<br><br>Empirical treatment for CRE infection, per day:$233	NS
**Zboromyrska, 2016[62]**	Technical staff (20 min per vial): $12<br><br>Microbiologist (10 min per vial): $12<br><br>Technical staff (15 min per sample): $9<br><br>Microbiologist (10 min per sample): $12		GeneXpert (per sample): $115	Broad-spectrum antibiotic, per day: $119<br><br>Narrow-spectrum antibiotic, per day: $84<br><br>Central venous catheter (average): $39<br><br>blood culture, per vial: $26<br><br>PET: $1202<br><br>Abdominal ultrasound: $189	GeneXpert and blood culture, per patient: $707

Notes: Costs were calculated in 2022 USDs. NS, Not stated. ARO, Antibiotic-resistant organism. ASTs, Antimicrobial Stewardship Teams. ICU= Intensive care unit. BSI, bloodstream infection. CDI, Clostridium difficile infections. CRE, carbapenem-resistant Enterobacteriaceae. CP, Current practice. DPO, Dual priming oligonucleotide. H. pylori, *Helicobacter pylori*. IPC, infection prevention and control. MRSA, methicillin-resistant Staphylococcus aureus. PCR, polymerase chain reaction. PCT, Procalcitonin. RUT, rapid urease test. UE, Union European. US, United States. WGS, whole-genome sequencing. US, United States. EU, European Union. MRSA= Methicillin-resistant Staphylococcus aureus.

Table SM10: Quality appraisal using Drummond's checklist.

First author	Q1	Q2	Q3	Q4	Q5	Q6	Q7	Q8	Q9	Q10	Score	Interpretation compared to studies' average score
**I. Pharmaceutical interventions** (N= 32 studies)												
Bianchini[3]	1	0	1	0	1	1	1	1	1	1	**8**	Average
Bolaños-Diaz[4]	1	1	1	1	0	1	1	1	1	1	**9**	Above average
Bounthavong[5]	1	1	1	1	0	1	0	1	1	1	**8**	Average
Bounthavong[6]	1	1	1	1	1	0	0	1	1	1	**8**	Average
Cara[7]	1	1	0	1	1	0	0	1	1	0	**6**	Below average
Collins[8]	1	1	1	1	1	1	1	1	1	1	**10**	Above average
De Cock[9]	1	1	0	0	1	1	0	1	1	1	**7**	Below average
De Cock[10]	1	1	1	0	1	0	0	1	1	1	**7**	Below average
Goudarzi[11]	1	1	1	0	1	1	1	1	1	1	**9**	Above average
Gutierrez[12]	1	1	1	1	0	1	1	1	1	0	**8**	Average
Kong[13]	1	1	1	1	1	1	1	1	1	1	**10**	Above average
Laohavaleeson[14]	1	1	1	1	1	1	0	1	1	1	**9**	Above average
Lin[15]	1	1	1	1	0	0	0	1	1	1	**7**	Below average
McKinnon[16]	1	1	1	1	1	1	0	1	0	1	**8**	Average
Mennini[17]	1	1	1	1	0	1	1	1	1	1	**9**	Above average
Mullins[18]	1	1	1	1	0	0	0	1	0	1	**6**	Below average
Niederman[19]	1	1	0	0	1	1	0	1	1	1	**7**	Below average
Patel[20]	1	1	1	1	1	1	0	1	1	0	**8**	Average
Patel[21]	1	1	1	1	1	1	0	1	1	1	**9**	Above average
Prabhu[22]	1	1	1	0	1	1	0	1	1	1	**8**	Average
Rubio-Terres[23]	1	1	1	1	1	1	1	1	1	0	**9**	Above average
Salas[24]	1	1	1	1	0	0	0	1	1	1	**7**	Below average
Schurmann[25]	1	1	1	1	0	1	0	1	1	1	**8**	Average
Simon[26]	1	1	1	1	1	1	1	1	1	1	**10**	Above average
Tan[27]	1	1	1	0	0	0	0	1	1	1	**6**	Below average
Varon[28]	1	1	1	1	1	1	0	1	1	1	**9**	Above average
Varon-Vega[29]	1	1	1	0	0	1	0	1	1	1	**7**	Below average
Vlachaki[30]	1	1	1	1	1	1	1	1	1	1	**10**	Above average
Vu[31]	1	1	1	1	1	1	0	1	1	1	**9**	Above average
Von Dach[32]	1	1	1	1	1	1	0	1	1	1	**9**	Above average
Wan[33]	1	1	1	1	1	0	0	1	1	1	**9**	Above average
Yang[34, 35]	1	1	1	1	1	1	1	1	1	0	**9**	Above average
**II. Non-pharmaceutical interventions** (N= 27 studies)												
Brown[36]	1	1	1	0	0	1	1	1	1	1	**8**	Average
Cho[37]	1	1	1	1	1	0	0	1	0	0	**6**	Below average
Dymond[38]	1	0	1	0	1	0	0	1	1	1	**6**	Below average
Gidengil[39]	1	1	1	1	1	1	1	1	1	1	**10**	Above average
Ho[40]	1	1	1	1	1	1	1	1	1	1	**10**	Above average
Hubben[41]	1	1	1	1	1	0	1	1	1	1	**9**	Above average
Jayaraman[42]	1	1	1	1	0	1	0	1	1	1	**8**	Average
Kang[43]	1	1	1	1	1	1	0	1	1	1	**9**	Above average
Lapointe-Shaw[44]	1	1	1	1	0	1	1	1	1	0	**8**	Average
Lee[45]	1	0	1	0	1	0	0	1	1	1	**6**	Below average
Lee[46]	1	1	1	0	0	1	0	1	1	1	**6**	Below average
Lee[47]	1	1	1	1	0	1	1	1	1	0	**8**	Average
Lin[48]	1	1	1	0	1	0	0	1	1	1	**7**	Below average
Luangasanatip[49]	1	1	1	1	1	1	1	1	1	1	**10**	Above average

Mac[50]	1	1	1	1	1	0	1	1	1	1	**9**	Above average
Murthy[51]	1	1	1	1	0	0	0	1	1	1	**7**	Below average
Nelson[52]	1	1	1	0	1	0	0	1	1	1	**7**	Below average
Nelson[53]	1	1	1	1	1	0	1	1	1	1	**9**	Above average
Nelson[54]	1	1	1	1	1	1	1	1	1	1	**10**	Above average
Penno[55]	1	0	1	1	0	1	0	1	1	1	**9**	Above average
Puzniak[56]	1	1	1	1	0	0	0	1	1	1	**7**	Below average
Robotham[57]	1	1	1	1	0	1	0	1	1	1	**8**	Average
Robotham[58]	1	1	1	1	1	1	0	1	1	1	**9**	Above average
Voermans[59]	1	1	1	0	1	0	0	1	1	1	**7**	Below average
You[60]	1	1	1	0	0	0	0	1	1	1	**6**	Below average
You[61]	1	1	1	1	0	1	1	1	1	1	**9**	Above average
Zboromyrska[62]	1	1	1	0	0	1	1	1	1	1	**9**	Above average
Average score among all studies					8.1							
Percentage from the total	100	93	95	71	63	66	39	100	95	86		

Notes: See Table SM5 for the full questions detailed. Q stands for question item from Drummond's checklist.[2, 64]

Table SM11: Prisma Checklist[65]

Section and Topic	Item #	Checklist item	Location where the item is reported
**TITLE**			
Title	1	Identify the report as a systematic review.	Title, first page
**ABSTRACT**			
Abstract	2	See the PRISMA 2020 for Abstracts checklist.	Abstract, first page
**INTRODUCTION**			
Rationale	3	Describe the rationale for the review in the context of existing knowledge.	Introduction
Objectives	4	Provide an explicit statement of the objective(s) or question(s) the review addresses.	Introduction, last paragraph
**METHODS**			
Eligibility criteria	5	Specify the inclusion and exclusion criteria for the review and how studies were grouped for the syntheses.	Methods, third paragraph
Information sources	6	Specify all databases, registers, websites, organisations, reference lists, and other sources searched or consulted to identify studies. Specify the date when each source was last searched or consulted.	Methods, third paragraph
Search strategy	7	Present the full search strategies for all databases, registers and websites, including any filters and limits used.	Supplementary material, Table SM2
Selection process	8	Specify the methods used to decide whether a study met the inclusion criteria of the review, including how many reviewers screened each record and each report retrieved, whether they worked independently, and, if applicable, details of automation tools used in the process.	Methods, paragraphs 3 and 4.
Data collection process	9	Specify the methods used to collect data from reports, including how many reviewers collected data from each report, whether they worked independently, any processes for obtaining or confirming data from study investigators, and, if applicable, details of automation tools used in the process.	Methods, paragraph 5.
Data items	10a	List and define all outcomes for which data were sought. Specify whether all results that were compatible with each outcome domain in each study were sought (e.g., for all measures, time points, analyses), and if not, the methods used to decide which results to collect.	Methods, paragraph 5.
	10b	List and define all other variables for which	Methods, paragraph 5.

Section and Topic	Item #	Checklist item	Location where the item is reported
		data were sought (e.g., participant and intervention characteristics, funding sources). Describe any assumptions made about any missing or unclear information.	
Study risk of bias assessment	11	Specify the methods used to assess the risk of bias in the included studies, including details of the tool(s) used, how many reviewers assessed each study and whether they worked independently, and if applicable, details of automation tools used in the process.	Methods, paragraph 6.
Effect measures	12	Specify for each outcome the effect measure(s) (e.g., risk ratio, mean difference) used in synthesizing or presenting results.	Methods, paragraph 5.
Synthesis methods	13a	Describe the processes used to decide which studies were eligible for each synthesis (e.g., tabulating the study intervention characteristics and comparing against the planned groups for each synthesis (item #5)).	Methods, paragraph 4.
	13b	Describe any methods required to prepare the data for presentation or synthesis, such as handling of missing summary statistics, or data conversions.	Methods, paragraph 5.
	13c	Describe any methods used to tabulate or visually display the results of individual studies and syntheses.	Methods, paragraph 5.
	13d	Describe any methods used to synthesize results and provide a rationale for the choice(s). If meta-analysis was performed, describe the model(s), method(s) to identify the presence and extent of statistical heterogeneity, and software package(s) used.	Methods, paragraph 5.
	13e	Describe any methods to explore possible causes of heterogeneity among study results (e.g., subgroup analysis, meta-regression).	Methods, paragraph 5.
	13f	Describe any sensitivity analyses conducted to assess the robustness of the synthesized results.	Methods, paragraph 5.
Reporting bias assessment	14	Describe any methods used to assess bias risk due to missing synthesis results (arising from reporting biases).	Methods, paragraph 6.
Certainty assessment	15	Describe any methods used to assess certainty (or confidence) in the body of evidence for an outcome.	Methods, paragraph 6.
**RESULTS**			
Study selection	16a	Describe the search and selection process	Results, first paragraph

Section and Topic	Item #	Checklist item	Location where the item is reported
		results, from the number of records identified in the search to the number of studies included in the review, ideally using a flow diagram.	
	16b	Cite studies that might appear to meet the inclusion criteria but which were excluded, and explain why they were excluded.	Results, first paragraph, and PRISMA chart
Study characteristics	17	Cite each included study and present its characteristics.	Supplementary Material
Risk of Bias in studies	18	Present assessments of risk of bias for each included study.	Supplementary Material and the last paragraph of the Results section
Results of individual studies	19	For all outcomes, present, for each study: (a) summary statistics for each group (where appropriate) and (b) an effect estimate and it's precision (e.g., confidence/credible interval), ideally using structured tables or plots.	Results
Results of syntheses	20a	For each synthesis, briefly summarise the characteristics and risk of bias among contributing studies.	Last paragraph of the Results section
	20b	Present results of all statistical syntheses conducted. If meta-analysis was done, present for each the summary estimate and its precision (e.g., confidence/credible interval) and measures of statistical heterogeneity. If comparing groups, describe the direction of the effect.	n/a
	20c	Present results of all investigations of possible causes of heterogeneity among study results.	Supplementary material
	20d	Present results of all sensitivity analyses conducted to assess the robustness of the synthesized results.	n/a
Reporting biases	21	Present assessments of risk of bias due to missing results (arising from reporting biases) for each synthesis assessed.	Last paragraph of the Results section
Certainty of evidence	22	Present assessments of certainty (or confidence) in the body of evidence for each outcome assessed.	n/a
**DISCUSSION**			
Discussion	23a	Provide a general interpretation of the results in the context of other evidence.	Discussion section
	23b	Discuss any limitations of the evidence included in the review.	Discussion section
	23c	Discuss any limitations of the review processes used.	Discussion section

Allel K, *et al. BMJ Glob Health* 2024; 9:e013205. doi: 10.1136/bmjgh-2023-013205

Section and Topic	Item #	Checklist item	Location where the item is reported
	23d	Discuss the implications of the results for practice, policy, and future research.	Discussion section
**OTHER INFORMATION**			
Registration and protocol	24a	Provide registration information for the review, including the register name and registration number, or state that the review was not registered.	Methods section, Prospero registration
	24b	Indicate where the review protocol can be accessed, or state that a protocol was not prepared.	Prospero protocol prepared
	24c	Describe and explain any amendments to information provided at registration or in the protocol.	n/a
Support	25	Describe sources of financial or non-financial support for the review, and the role of the funders or sponsors in the review.	n/a
Competing interests	26	Declare any competing interests of review authors.	n/a
Availability of data, code, and other materials	27	The report which of the following are publicly available and where they can be found: template data collection forms; data extracted from included studies; data used for all analyses; analytic code; any other materials used in the review.	Data are provided in Excel (https://bit.ly/SR_amrCEingredients).

Notes: n/a= not applicable.

References

1. Tacconelli E, Carrara E, Savoldi A, Harbarth S, Mendelson M, Monnet DL, et al. Discovery, research, and development of new antibiotics: the WHO priority list of antibiotic-resistant bacteria and tuberculosis. Lancet Infect Dis. 2018;18(3):318-27. Epub 2017/12/26. doi: 10.1016/s1473-3099(17)30753-3. PubMed PMID: 29276051.

2. Drummond MF, Sculpher MJ, Claxton K, Stoddart GL, Torrance GW. Methods for the Economic Evaluation of Health Care Programmes. Oxford: Oxford: Oxford University Press; 2015.

3. Bianchini ML, Jeffres MN, Campbell JD. Cost-Effectiveness Analysis of New Beta-Lactam Beta-Lactamase Inhibitor Antibiotics Versus Colistin for the Treatment of Carbapenem-Resistant Infections. Hospital Pharmacy. 2022;57(1):93-100.

4. Bolaños-Díaz R, Angles-Yanqui E, Pérez-Lazo G, Sanabria-Montañez C. Cost-effectiveness of ceftazidime/avibactam for infections due to carbapenem-resistant bacteria in Peru. Journal of Pharmaceutical Health Services Research. 2022;13(1):2-8.

5. Bounthavong M, Hsu D, Okamoto M. Cost-effectiveness analysis of linezolid vs. vancomycin in treating methicillin-resistant Staphylococcus aureus complicated skin and soft tissue infections using a decision analytic model. International journal of clinical practice. 2009;63(3):376-86.

6. Bounthavong M, Zargarzadeh A, Hsu DI, Vanness DJ. Cost-effectiveness analysis of linezolid, daptomycin, and vancomycin in methicillin-resistant Staphylococcus aureus: complicated skin and skin structure infection using Bayesian methods for evidence synthesis. Value in Health. 2011;14(5):631-9.

7. Cara AKS, Zaidi STR, Suleman F. Cost-effectiveness analysis of low versus high dose colistin in the treatment of multi-drug resistant pneumonia in Saudi Arabia. International Journal of Clinical Pharmacy. 2018;40:1051-8.

8. Collins CD, Schwemm AK. Linezolid versus vancomycin in the empiric treatment of nosocomial pneumonia: a cost-utility analysis incorporating results from the ZEPHyR trial. Value in health. 2015;18(5):614-21.

9. De Cock E, Sorensen S, Levrat F, Besnier JM, Dupon M, Guery B, et al. Cost-effectiveness of linezolid versus vancomycin for hospitalized patients with complicated skin and soft-tissue infections in France. Med Mal Infect. 2009;39(5):330-40. doi: https://dx.doi.org/10.1016/j.medmal.2009.01.005. PubMed PMID: 19304423.

10. De Cock E, Krueger WA, Sorensen S, Baker T, Hardewig J, Duttagupta S, et al. Cost-effectiveness of linezolid vs vancomycin in suspected methicillin-resistant Staphylococcus aureus nosocomial pneumonia in Germany. Infection. 2009;37(2):123-32. doi: https://dx.doi.org/10.1007/s15010-008-8046-7. PubMed PMID: 19277465.

11. Goudarzi Z, Danayi F, Keshavarz K, Gholami A. Cost-effectiveness analysis of ceftazidime avibactam versus colistin in carbapenem-resistant enterobacteriaceae in Iran. Cost Effectiveness and Resource Allocation. 2023;21(1):45.

12. Gutiérrez A, Fandino C. Cost-effectiveness of ceftazidime/avibactam versus colistin+ meropenem for treatment of carbapenemic-resistant enterobacteria infections in Chile. Revista Chilena de Infectologia: Organo Oficial de la Sociedad Chilena de Infectologia. 2021;38(1):7-14.

13. Kong W, Yang X, Shu Y, Li S, Song B, Yang K. Cost-effectiveness analysis of ceftazidime-avibactam as definitive treatment for treatment of carbapenem-resistant Klebsiella pneumoniae bloodstream infection. Frontiers in Public Health. 2023;11:1118307.

14. Laohavaleeson S, Barriere SL, Nicolau DP, Kuti JL. Cost-effectiveness of telavancin versus vancomycin for treatment of complicated skin and skin structure infections. Pharmacotherapy. 2008;28(12):1471-82. doi: https://dx.doi.org/10.1592/phco.28.12.1471. PubMed PMID: 19025428.

15. Lin P-C, Wang BC, Kim R, Magyar A, Lai C-C, Yang Y-W, et al. Estimating the cost-effectiveness of linezolid for the treatment of methicillin-resistant Staphylococcus aureus nosocomial pneumonia in Taiwan. Journal of Microbiology, Immunology and Infection. 2016;49(1):46-51.

16. McKinnon PS, Sorensen SV, Liu LZ, Itani KM. Impact of linezolid on economic outcomes and determinants of cost in a clinical trial evaluating patients with MRSA complicated skin and soft-tissue infections. Ann Pharmacother. 2006;40(6):1017-23. PubMed PMID: 16720705.

17. Mennini FS, Gori M, Vlachaki I, Fiorentino F, Malfa PL, Urbinati D, et al. Cost-effectiveness analysis of Vaborem in Carbapenem-resistant Enterobacterales (CRE)-Klebsiella pneumoniae infections in Italy. Health Economics Review. 2021;11(1):1-10.

18. Daniel Mullins C, Kuznik A, Shaya FT, Obeidat NA, Levine AR, Liu LZ, et al. Cost-effectiveness analysis of linezolid compared with vancomycin for the treatment of nosocomial pneumonia caused by methicillin-resistant Staphylococcus aureus. Clin Ther. 2006;28(8):1184-98. doi: https://dx.doi.org/10.1016/j.clinthera.2006.08.016. PubMed PMID: 16982296.

19. Niederman MS, Chastre J, Solem CT, Wan Y, Gao X, Myers DE, et al. Health economic evaluation of patients treated for nosocomial pneumonia caused by methicillin-resistant Staphylococcus aureus: secondary analysis of a multicenter randomized clinical trial of vancomycin and linezolid. Clin Ther. 2014;36(9):1233-43.e1. doi: https://dx.doi.org/10.1016/j.clinthera.2014.06.029. PubMed PMID: 25066668.

20. Patel DA, Michel A, Stephens J, Weber B, Petrik C, Charbonneau C. An economic model to compare linezolid and vancomycin for the treatment of confirmed methicillin-resistant Staphylococcus aureus nosocomial pneumonia in Germany. Infect. 2014;7:273-80. doi: https://dx.doi.org/10.2147/IDR.S68658. PubMed PMID: 25368526.

21. Patel DA, Shorr AF, Chastre J, Niederman M, Simor A, Stephens JM, et al. Modeling the economic impact of linezolid versus vancomycin in confirmed nosocomial pneumonia caused by methicillin-resistant Staphylococcus aureus. Critical Care (London, England). 2014;18(4):R157. PubMed PMID: rayyan-844244224.

22. Prabhu V, Foo J, Ahir H, Sarpong E, Merchant S. Cost-effectiveness of ceftolozane/tazobactam plus metronidazole compared with piperacillin/tazobactam as empiric therapy for the treatment of complicated intra-abdominal infections based on the in-vitro surveillance of bacterial isolates in the UK. J Med Econ. 2017;20(8):840-9. doi: https://dx.doi.org/10.1080/13696998.2017.1333960. PubMed PMID: 28532194.

23. Terrés CR, Rodríguez DR, Oró NM, Grau S. Análisis farmacoeconómico del tratamiento de la bacteriemia por Staphylococcus aureus resistente a meticilina con daptomicina y vancomicina. Revista Española de Quimioterapia. 2012;25(4):283-92.

24. del Diego Salas J, Orly de Labry Lima A, Espin Balbino J, Bermudez Tamayo C, Fernandez-Crehuet Navajas J. An economic evaluation of two interventions for the prevention of post-surgical infections in cardiac surgery. Rev. 2016;31(1):27-33. doi: https://dx.doi.org/10.1016/j.cali.2015.08.007. PubMed PMID: 26602758.

25. Schurmann D, Sorensen SV, De Cock E, Duttagupta S, Resch A. Cost-effectiveness of linezolid versus vancomycin for hospitalised patients with complicated skin and soft-tissue infections in Germany. Eur J Health Econ. 2009;10(1):65-79. doi: https://dx.doi.org/10.1007/s10198-008-0104-7. PubMed PMID: 18437437.

26. Simon M, Sfeir MM, Calfee DP, Satlin MJ. Cost-effectiveness of ceftazidime-avibactam for treatment of carbapenem-resistant Enterobacteriaceae bacteremia and pneumonia. Antimicrobial Agents and Chemotherapy. 2019;63(12):10.1128/aac. 00897-19.

27. Tan SC, Wang X, Wu B, Kang H, Li Q, Chen Y, et al. Cost-effectiveness of linezolid versus vancomycin among patients with methicillin-resistant Staphylococcus aureus confirmed nosocomial pneumonia in China. Value in Health Regional Issues. 2014;3:94-100.

28. Varón F, Londoño D, Álvarez C, Taborda A, Prieto V. Costo-efectividad de linezolid comparado con vancomicina en el manejo de la neumonía asociada a ventilación mecánica en Colombia. Infectio. 2014;18(4):143-52.

29. Varón-Vega F, Lemos E, Castaño GN, Reyes J. Cost-utility analysis of ceftazidime-avibactam versus colistin-meropenem in the treatment of infections due to Carbapenem-resistant Klebsiella pneumoniae in Colombia. Expert Review of Pharmacoeconomics & Outcomes Research. 2022;22(2):235-40.

30. Vlachaki I, Zinzi D, Falla E, Mantopoulos T, Guy H, Jandu J, et al. Cost-effectiveness analysis of vaborem for the treatment of carbapenem-resistant Enterobacteriaceae-Klebsiella pneumoniae carbapenemase (CRE-KPC) infections in the UK. The European Journal of Health Economics. 2021:1-13.

31. Vu M, Smith KJ, Aspinall SL, Clancy CJ, Buehrle DJ. Exploratory cost-effectiveness analysis for treatment of methicillin-resistant Staphylococcus aureus bloodstream infections: is linezolid or daptomycin favored over vancomycin? Clinical Drug Investigation. 2021;41:885-94.

32. Von Dach E, Morel C, Murthy A, Pagani L, Macedo-Vinas M, Olearo F, et al. Comparing the cost-effectiveness of linezolid to trimethoprim/sulfamethoxazole plus rifampicin for the treatment of methicillin-resistant Staphylococcus aureus infection: a healthcare system perspective. Clinical Microbiology and Infection. 2017;23(9):659-66.

33. Wan Y, Li Q, Chen Y, Haider S, Liu S, Gao X. Economic evaluation among Chinese patients with nosocomial pneumonia caused by methicillin-resistant Staphylococcus aureus and treated with linezolid or vancomycin: a secondary, post-hoc analysis based on a phase 4 clinical trial study. J Med Econ. 2016;19(1):53-62.

34. Yang J, Naik J, Massello M, Ralph L, Dillon RJ. Cost-Effectiveness of Imipenem/Cilastatin/Relebactam Compared with Colistin in Treatment of Gram-Negative Infections Caused by Carbapenem-Non-Susceptible Organisms. Infectious Diseases & Therapy. 2022;25:25. doi: https://dx.doi.org/10.1007/s40121-022-00607-x. PubMed PMID: 35334080.

35. Yang J, Naik J, Massello M, Ralph L, Dillon RJ. Correction to: Cost-Effectiveness of Imipenem/Cilastatin/Relebactam Compared with Colistin in Treatment of Gram-Negative Infections Caused by Carbapenem-Non-Susceptible Organisms. Infectious Diseases and Therapy. 2022;11(4):1459.

36. Brown J, Paladino JA. Impact of rapid methicillin-resistant Staphylococcus aureus polymerase chain reaction testing on mortality and cost effectiveness in hospitalized patients with bacteraemia. Pharmacoeconomics. 2010;28(7):567-75.

37. Cho JH, Jeon SR, Kim HG, Jin SY, Park S. Cost-effectiveness of a tailored Helicobacter pylori eradication strategy based on the presence of a 23 S ribosomal RNA point mutation that causes clarithromycin resistance in Korean patients. Journal of gastroenterology and hepatology. 2019;34(4):700-6.

38. Dymond A, Davies H, Mealing S, Pollit V, Coll F, Brown NM, et al. Genomic surveillance of methicillin-resistant Staphylococcus aureus: a mathematical early modeling study of cost-effectiveness. Clin Infect Dis. 2020;70(8):1613-9.

39. Gidengil CA, Gay C, Huang SS, Platt R, Yokoe D, Lee GM. Cost-effectiveness of strategies to prevent methicillin-resistant Staphylococcus aureus transmission and infection in an intensive care unit. infection control & hospital epidemiology. 2015;36(1):17-27.

40. Ho K-w, Ng W-t, Ip M, You JH. Active surveillance of carbapenem-resistant Enterobacteriaceae in intensive care units: Is it cost-effective in a nonendemic region? Am J Infect Control. 2016;44(4):394-9.

41. Hubben G, Bootsma M, Luteijn M, Glynn D, Bishai D, Bonten M, et al. Modelling the costs and effects of selective and universal hospital admission screening for methicillin-resistant Staphylococcus aureus. PloS one. 2011;6(3):e14783.

42. Jayaraman SP, Jiang Y, Resch S, Askari R, Klompas M. Cost-effectiveness of a model infection control program for preventing multi-drug-resistant organism infections in critically ill surgical patients. Surgical Infections. 2016;17(5):589-95.

43. Kang J, Mandsager P, Biddle AK, Weber DJ. Cost-effectiveness analysis of active surveillance screening for methicillin-resistant Staphylococcus aureus in an academic hospital setting. Infection Control & Hospital Epidemiology. 2012;33(5):477-86.

44. Lapointe-Shaw L, Voruganti T, Kohler P, Thein H-H, Sander B, McGeer A. Cost-effectiveness analysis of universal screening for carbapenemase-producing Enterobacteriaceae in hospital inpatients. European Journal of Clinical Microbiology & Infectious Diseases. 2017;36(6):1047-55.

45. Lee TA, Hacek DM, Stroupe KT, Collins SM, Peterson LR. Three surveillance strategies for vancomycin-resistant enterococci in hospitalized patients: detection of colonization efficiency and a cost-effectiveness model. Infection Control & Hospital Epidemiology. 2005;26(1):39-46.

46. Lee BY, Tsui BY, Bailey RR, Smith KJ, Muder RR, Lewis GJ, et al. Should vascular surgery patients be screened preoperatively for methicillin-resistant Staphylococcus aureus? Infection Control & Hospital Epidemiology. 2009;30(12):1158-65.

47. Lee BY, Bailey RR, Smith KJ, Muder RR, Strotmeyer ES, Lewis GJ, et al. Universal methicillin-resistant Staphylococcus aureus (MRSA) surveillance for adults at hospital admission: an economic model and analysis. Infection Control & Hospital Epidemiology. 2010;31(6):598-606.

48. Lin G, Tseng KK, Gatalo O, Martinez DA, Hinson JS, Milstone AM, et al. Cost-effectiveness of carbapenem-resistant Enterobacteriaceae (CRE) surveillance in Maryland. Infection Control & Hospital Epidemiology. 2021:1-9.

49. Luangasanatip N, Hongsuwan M, Lubell Y, Limmathurotsakul D, Srisamang P, Day N, et al. Cost-effectiveness of interventions to improve hand hygiene in healthcare workers in middle-income hospital settings: a model-based analysis. J Hosp Infect. 2018;100(2):165-75.

50. Mac S, Fitzpatrick T, Johnstone J, Sander B. Vancomycin-resistant enterococci (VRE) screening and isolation in the general medicine ward: a cost-effectiveness analysis. Antimicrobial Resistance & Infection Control. 2019;8(1):1-10.

51. Murthy A, De Angelis G, Pittet D, Schrenzel J, Uckay I, Harbarth S. Cost-effectiveness of universal MRSA screening on admission to surgery. Clinical microbiology and infection. 2010;16(12):1747-53.

52. Nelson R, Samore M, Smith K, Harbarth S, Rubin M, Program CPE. Cost-effectiveness of adding decolonization to a surveillance strategy of screening and isolation for methicillin-resistant Staphylococcus aureus carriers. Clinical microbiology and infection. 2010;16(12):1740-6.

53. Nelson RE, Stevens VW, Khader K, Jones M, Samore MH, Evans ME, et al. Economic analysis of Veterans Affairs initiative to prevent methicillin-resistant Staphylococcus aureus infections. American journal of preventive medicine. 2016;50(5):S58-S65.

54. Nelson RE, Goto M, Samore MH, Jones M, Stevens VW, Evans ME, et al. Expanding an economic evaluation of the Veterans Affairs (VA) methicillin-resistant Staphylococcus aureus (MRSA) prevention initiative to include prevention of infections from other pathogens. Clin Infect Dis. 2021;72(Supplement_1):S50-S8.

55. Penno EC, Baird SJ, Crump JA. Cost-effectiveness of surveillance for bloodstream infections for sepsis management in low-resource settings. The American Journal of Tropical Medicine and Hygiene. 2015;93(4):850.

56. Puzniak LA, Gillespie KN, Leet T, Kollef M, Mundy LM. A Cost-Benefit Analysis of Gown Use in Controlling Vancomycin-Resistant Enterococcus Transmission Is It Worth the Price? Infection Control & Hospital Epidemiology. 2004;25(5):418-24.

57. Robotham JV, Graves N, Cookson BD, Barnett AG, Wilson JA, Edgeworth JD, et al. Screening, isolation, and decolonisation strategies in the control of meticillin resistant Staphylococcus aureus in intensive care units: cost effectiveness evaluation. Bmj. 2011;343.

58. Robotham JV, Deeny SR, Fuller C, Hopkins S, Cookson B, Stone S. Cost-effectiveness of national mandatory screening of all admissions to English National Health Service hospitals for meticillin-resistant Staphylococcus aureus: a mathematical modelling study. The Lancet Infectious Diseases. 2016;16(3):348-56.

59. Voermans AM, Mewes JC, Broyles MR, Steuten LM. Cost-effectiveness analysis of a procalcitonin-guided decision algorithm for antibiotic stewardship using real-world US hospital data. Omics: a journal of integrative biology. 2019;23(10):508-15.

60. You JH, Chan C, Wong M, Ip M. Active surveillance and decolonization of methicillin-resistant Staphylococcus aureus on admission to neonatal intensive care units in Hong Kong: a cost-effectiveness analysis. Infection Control & Hospital Epidemiology. 2012;33(10):1024-30.

61. You JH, Li H-k, Ip M. Surveillance-guided selective digestive decontamination of carbapenem-resistant Enterobacteriaceae in the intensive care unit: A cost-effectiveness analysis. Am J Infect Control. 2018;46(3):291-6.

62. Zboromyrska Y, De la Calle C, Soto M, Sampietro-Colom L, Soriano A, Alvarez-Martínez MJ, et al. Rapid diagnosis of staphylococcal catheter-related bacteraemia in direct blood samples by real-time PCR. PLoS One. 2016;11(8):e0161684.

63. Online RB. IBM Micromedex [database online]. 2020.

64. Drummond MF, O'Brien BJ, Torrance GW, Stoddart GL. Methods for the Economic Evaluation of Health Care Programmes. Oxford: Oxford University Press; 1997.

65. Page MJ, McKenzie JE, Bossuyt PM, Boutron I, Hoffmann TC, Mulrow CD, et al. The PRISMA 2020 statement: an updated guideline for reporting systematic reviews. BMJ. 2021;372:n71. doi: 10.1136/bmj.n71.
